# An orally deliverable Co_3_O_4_@MMT nanozyme platform for inflammatory bowel disease via ROS scavenging, barrier repair, and mucosal homeostasis regulation

**DOI:** 10.1016/j.mtbio.2026.103310

**Published:** 2026-06-01

**Authors:** Yinxi Li, Qinxuan Zhou, Yuxuan She, Bin Lu, Mengting Wu, Tianhao Chen, Pengcheng Ye, Li Yu, Wenzhao Liu, Hui Deng, Xiaowen Hu

**Affiliations:** a School and Hospital of Stomatology, Wenzhou Medical University, Wenzhou, Zhejiang, 325001, China; b The Affiliated Stomatological Hospital of Chongqing Medical University, Chongqing Key Laboratory of Oral Diseases, Chongqing Municipal Key Laboratory of Oral Biomedical Engineering of Higher Education, Chongqing Municipal Health Commission Key Laboratory of Oral Biomedical Engineering, Chongqing, 401147, China

## Abstract

Inflammatory bowel disease (IBD) is a chronic gastrointestinal disorder characterized by a self-perpetuating pathological loop, where excessive reactive oxygen species (ROS) induce mucosal inflammation, which in turn drives further ROS production. This oxidative-inflammatory cycle leads to intestinal epithelial barrier disruption and gut microbiota dysbiosis. Current immunosuppressive therapies primarily target downstream inflammation, while fail to address the foundational oxidative stress and achieve complete mucosal healing. To bridge this gap, we developed an orally deliverable nanozyme platform by anchoring cobalt oxide (Co_3_O_4_) nanoparticles onto montmorillonite nanosheets (Co_3_O_4_@MMT). This design leverages the Co^2+^/Co^3+^ redox cycling within Co_3_O_4_ for efficient multi-enzymatic ROS scavenging. Furthermore, transcriptomic analysis and literature evidence suggest that the released Co^2+^ ions can activate the HIF-1α pathway—a key regulator of epithelial repair. The MMT component protects the nanozyme from gastrointestinal degradation and facilitates its electrostatic accumulation at inflamed colonic sites. In murine colitis models, oral Co_3_O_4_@MMT treatment effectively alleviated disease severity, suppressed inflammation, restored barrier integrity, and rebalanced gut microbiota. This work presents an integrated oral strategy that concurrently targets oxidative stress, inflammation, and barrier restoration, offering a comprehensive therapeutic approach for IBD.

## Highlights

• An orally deliverable Co_3_O_4_@MMT nanozyme platform was developed by anchoring cobalt oxide nanoparticles onto exfoliated montmorillonite nanosheets.
• MMT serves as a protective and targeting carrier, shielding Co_3_O_4_ from gastrointestinal degradation and enabling electrostatic accumulation at inflamed colonic mucosa.
• The Co^2+^/Co^3+^ redox cycling endows Co_3_O_4_@MMT with potent multi-enzymatic (SOD, CAT, .•OH) ROS-scavenging activity, effectively breaking the oxidative–inflammatory cycle in colitis.
• Beyond ROS clearance, Co_3_O_4_@MMT actively promotes intestinal barrier repair by upregulating tight junction proteins (Claudin-1, Occludin, ZO-1) and activating HIF-1 signaling pathway.
• Oral administration of Co_3_O_4_@MMT achieves comprehensive therapeutic efficacy in DSS-induced colitis mice, simultaneously alleviating oxidative stress, suppressing inflammation, restoring mucosal barrier integrity, and rebalancing gut microbiota.

## Graphical abstract

**Figure:**
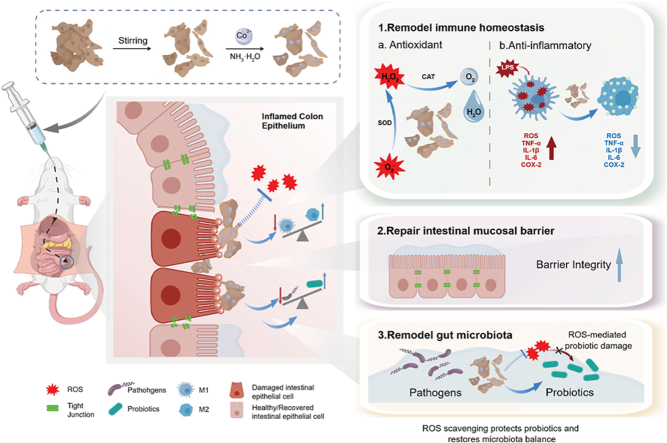

## 1 Introduction

**Scheme 1:**
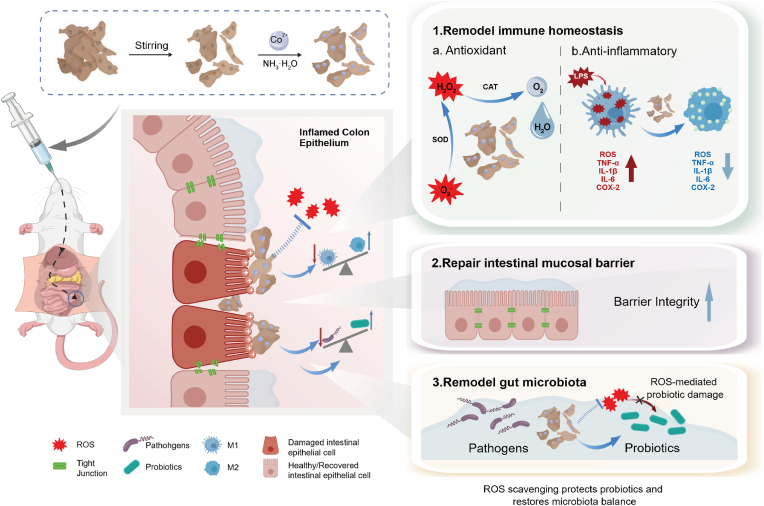
Schematic illustration of the multifunctional therapeutic mechanism of Co_3_O_4_@MMT in colitis, through ROS scavenging, anti-inflammation, intestinal barrier repair, and gut microbiota remodeling. Created with BioGDP.com [9].

Inflammatory bowel disease (IBD), encompassing ulcerative colitis and Crohn's disease, is a chronic, relapsing inflammatory disorder of the gastrointestinal tract [1,2]. A central driver of its pathogenesis is the excessive generation of reactive oxygen species (ROS) [3], which initiates a self-amplifying cycle [4]: ROS directly provoke mucosal inflammation [5], activating pro-inflammation signaling pathways, such as nuclear NF-κB [6]. And the ensuing inflammatory cascade further stimulates ROS production [7]. This oxidative-inflammatory interplay damages epithelial cells, compromises tight junctions, disrupts gut microbiota homeostasis, and perpetuates disease activity and tissue injury [8] (see Scheme 1).

Existing pharmacological interventions for IBD, such as corticosteroids, aminosalicylates, and biologics, primarily target downstream inflammatory mediators [10]. However, these agents seldom neutralize the upstream oxidative trigger, and long-term use is often associated with systemic side effects, including immunosuppression and metabolic disturbances [11]. Crucially, they frequently fail to achieve complete mucosal healing [12]. Therefore, an unmet therapeutic need exists for strategies capable of breaking the ROS-inflammation cycle while actively promoting epithelial repair and immune homeostasis.

Nanozymes—nanomaterials with enzyme-mimetic activities—have emerged as promising candidates for ROS scavenging due to their high stability, tunable catalysis, and cost-effectiveness [13]. Among them, cobalt-based nanozymes (Co_3_O_4_) exhibit potent multi-enzymatic antioxidant properties [14], attributed to their reversible Co^2+^/Co^3+^ redox couples that facilitate efficient electron transfer and catalytic degradation of various ROS [15,16]. Beyond catalysis, cobalt ions (Co^2+^) released from Co_3_O_4_ have been reported to modulate cellular signaling pathways critical for tissue repair [17]. Notably, Co^2+^ can stabilize the hypoxia-inducible factor-1α(HIF-1α), thereby activating its downstream signaling pathway [18]—a master regulator of epithelial cell survival, proliferation, and barrier function [12,19,20]. Therefore, we hypothesized that Co_3_O_4_ might alleviate colitis not only through its antioxidant and anti-inflammatory properties but also via the modulation of key signaling pathways, such as those related to cellular hypoxia response, which have been implicated in mucosal repair [[21], [22], [23]]. This dual capability—ROS clearance and potential HIF-1α-mediated repair—makes cobalt nanozymes a compelling therapeutic candidate for IBD.

Nevertheless, the oral delivery of bare Co_3_O_4_ nanoparticles is hindered by gastrointestinal degradation, potential metal ion leakage that leads to systemic toxicity [24], and reduced catalytic activity in the digestive tract. And sustained-release material systems play a critical role in IBD treatment by prolonging drug retention in the inflamed colon, reducing systemic exposure, and enabling long-lasting therapeutic effects with fewer administrations [25]. To overcome these barriers, we introduced montmorillonite (MMT), a biocompatible clay mineral with a high surface area and negative surface charge [26,27]. And conventional nanospheres for IBD therapy are typically designed for efficient cellular uptake and intracellular delivery [28,29]. In contrast, two-dimensional nanosheets such as exfoliated MMT possess a high aspect ratio and negatively charged surface, which enable prolonged adhesion to the damaged colonic mucosa and enhanced local retention of therapeutic agents. In our design, MMT serves two essential functions: (1) acting as a protective matrix that shields Co_3_O_4_ from acidic and enzymatic degradation, preserving its catalytic integrity, and (2) enabling targeted delivery through electrostatic adhesion to positively charged proteins overexpressed on inflamed colonic mucosa [30].

By integrating the ROS-scavenging and potential repair-promoting functions of Co_3_O_4_ with the protective and targeting capabilities of MMT, we engineered an orally deliverable Co_3_O_4_@MMT nanoplatform. We hypothesize that this system will not only intercept the oxidative-inflammatory cycle but also promote epithelial regeneration and barrier restoration, thereby addressing multiple pathological facets of IBD. In this study, we detail the synthesis, characterization, and comprehensive evaluation of Co_3_O_4_@MMT, demonstrating its efficacy as a multifunctional oral therapy for colitis.

## 2 Materials and methods

### 2.1 Materials

The chemicals MMT, Co(NO_3_)_2_·6H_2_O, and NH_3_·H_2_O were supplied by Shanghai Macklin Biochemical Co., Ltd. (China). For biological assays, we procured the SOD (NBT method), EdU proliferation, and TUNEL apoptosis, BCA protein and JC-1 assay kits, along with DAPI and Hoechst 33,342, from Beyotime Biotechnology (Shanghai, China). Suzhou Grace Biotechnology (China) provided the assay kits for catalase (CAT) activity, hydroxyl free radical scavenging, SOD and GSH-Px. Mito-SOX was sourced from Thermo Fisher Scientific (Waltham, MA, USA). We obtained simulated gastric fluid (SGF), simulated intestinal fluid (SIF), and dextran sulfate sodium (DSS) salt from Shanghai Yuanye Bio-Technology, while lipopolysaccharide (LPS) and hydrogen peroxide (H_2_O_2_), FITC and FITC-Dextran were acquired from Shanghai Aladdin Bio-Chem.

Regarding cell culture, DMEM was sourced from Cytiva (USA) and fetal bovine serum (FBS) was purchased from Procell Life Science & Technology (Wuhan, China). The RAW264.7 mouse macrophages and human Caco-2 cell lines were both provided by the ATCC (USA). For molecular analysis, Nanjing Vazyme Biotech (China) supplied the RNA extraction and reverse transcription kits, SYBR Green qPCR master mix, and the Live/Dead Cell Double Staining Kit. The CCK-8 assay was obtained from APExBIO (USA; Cat. No. K1018). Antibodies against β-actin, VEGFA, and p-p65 were purchased from Abclonal (Wuhan, China); GLUT1 from Cell Signaling Technology (Danvers, MA, USA); HIF-1α, Claudin-1, and Occludin from Proteintech (Rosemont, IL, USA); ZO-1 from HUABIO (Hangzhou, China); and p-STAT1 and p-STAT6 from ZenBio (Durham, NC, USA) and Affinity Biosciences (USA), respectively. Additionally, 4% paraformaldehyde and DCFH-DA were purchased from Beijing Solarbio, while antibodies against iNOS and CD206 were supplied by Beijing Bioss. All experimental reagents were utilized as received from the manufacturers unless otherwise specified.

### 2.2 Preparation and characteristic of Co_3_O_4_@MMT

The Co_3_O_4_@MMT nanocomposites were fabricated using an in-situ growth technique on MMT templates. Initially, we dispersed varying amounts of MMT powder (0.45, 0.20, or 0.1167 g) into 100 mL of deionized water and maintained stirring for a 48-h period. Subsequently, 10 mL of an aqueous solution containing 182 mg of Co(NO_3_)_2_·6H_2_O was introduced into the MMT suspension, followed by heating in a water bath with continuous agitation. To trigger the synthesis, we rapidly injected 0.8 mL of NH_3_·H_2_O (28%) and allowed the mixture to stir vigorously for 3 h.

The resulting Co_3_O_4_@MMT was then harvested via centrifugation and purified by washing three times with deionized water. These final products were either kept in a redispersed state in water for ongoing tests or preserved as a freeze-dried powder. Based on the initial mass ratios of Co_3_O_4_ to MMT, the samples were designated as Co_3_O_4_@MMT (1:9), (2:8), and (3:7). As a comparative control, free Co_3_O_4_ nanoparticles were prepared following the same protocol but without the addition of MMT. For labeling, a 50 μL FITC solution (1 mg/mL) was combined with 2 mL of Co_3_O_4_@MMT (0.5 mg/mL) and left to react at room temperature for 4 h. This was followed by exhaustive dialysis against ultrapure water to eliminate unbound dye, resulting in FITC-Co_3_O_4_@MMT. To characterize the materials, ICP-OES (Agilent 5110) was employed to determine elemental concentrations, while DLS (NanoTM 90) was used to evaluate hydrodynamic diameters and zeta potentials. Crystalline phases were identified by XRD (X'Pert PRO MPD), and Raman spectroscopy (LabRam HR Evolution) was performed to probe molecular vibrations and structural features. Additional structural insights were obtained through FT-IR (Nicolet iS 10) and XPS (Thermo Kalpha) analyses.

### 2.3 Evaluation of catalytic antioxidant performance and gastrointestinal stability

To evaluate the antioxidant enzyme-mimetic properties of Co_3_O_4_@MMT, we assessed its SOD-like activity, CAT-like activity, and hydroxyl radical (•OH) scavenging capacity. SOD-mimetic and •OH scavenging performances were measured using commercial assay kits following the manufacturer's instructions. In these assays, serial concentrations of Co_3_O_4_, MMT, and Co_3_O_4_@MMT (100-1000 μg/mL) were introduced into the corresponding reaction mixtures. Absorbance at 450 nm was recorded to determine SOD-like activity, while •OH scavenging ability was quantified at 510 nm using a microplate reader (SpectraMax M5). CAT-like activity was evaluated separately by monitoring oxygen generation using a dissolved oxygen meter (JPB-607 A, INESA, Shanghai, China). Briefly, varying concentrations of each sample were added to the reaction system, and the rate of oxygen production was measured in real time. The steady-state kinetics of CAT-like activity were assayed by varying H_2_O_2_ concentration (500 -5000 mg/L) in 5 mL of PBS containing Co_3_O_4_ (100 μg/mL) or Co_3_O_4_@MMT (500 μg/mL). Initial reaction velocity (*v*_0_) was calculated from the O_2_ generation within the first 5 s using a dissolved oxygen meter. The obtained *v*_0_ values were then plotted against H_2_O_2_ concentrations and fitted to the Michaelis-Menten equation to obtain the kinetic parameters (*K*_m_ and *V*_max_).

To investigate how the gastrointestinal environment affects the structural and functional resilience of Co_3_O_4_@MMT, we blended the material (1 mg/mL) with either simulated gastric fluid (SGF) or simulated intestinal fluid (SIF) at a 5:4 vol ratio. These preparations were incubated for 2 h at 37 °C. Following this treatment, we harvested the samples via centrifugation and purified them through three successive PBS washes. [31]. Structural integrity was monitored by analyzing zeta potential shifts using DLS. Furthermore, we assessed functional stability by measuring the residual SOD-like, CAT-like, and OH scavenging activities of the samples after the digestion process to confirm their sustained catalytic potency.

### 2.4 Cell viability and cellular uptake

For cell maintenance, we cultured RAW264.7 macrophages in DMEM supplemented with 10% fetal bovine serum (FBS), while Caco-2 cells were grown in their specific recommended medium. To simulate pathological conditions in vitro, we established inflammation and oxidative stress models by exposing RAW 264.7 cells to LPS (2 μg/mL) and Caco-2 cells to H_2_O_2_ (64 μM) for 24 h.

We assessed cell viability using the CCK-8 assay. Specifically, both cell lines were seeded into 96-well plates at a density of 4 × 10^4^ cells per well and allowed to adhere for 24 h. Subsequently, the supernatant was replaced with fresh medium containing varying concentrations (0.25 to 10 μg/mL) of MMT, Co_3_O_4_, or Co_3_O_4_@MMT. Following an additional 24-h incubation, we introduced 100 μL of CCK-8 working solution into each well and maintained the plates at 37 °C for 1 h. We then recorded the optical density at 450 nm, calculating the survival rate relative to the untreated control group.

To visually confirm biocompatibility, we performed live/dead staining. RAW264.7 and Caco-2 cells were inoculated into 24-well plates (1 × 10^5^ cells per well) and subjected to the aforementioned treatment conditions. After rinsing the cells with PBS, we incubated them with a mixture of Calcein-AM and propidium iodide at 37 °C for 30 min. Finally, we captured fluorescence images using a ZEISS AxioObserver3 inverted microscope. In these images, viable cells were identified by green fluorescence, whereas dead cells were characterized by red fluorescence.

To evaluate the cellular uptake of Co_3_O_4_@MMT, RAW 264.7 and Caco-2 cells were seeded into 24-well plates at a density of 1 × 10^5^ cells per well and cultured for 24 h. FITC-Co_3_O_4_@MMT (50 μg/mL) was then added and incubated for an additional 24 h. Subsequently, the medium was discarded, and the cells were washed three times with PBS and stained with Hoechst 33,342. The intracellular uptake of the material was observed under a fluorescence inverted microscope, and the fluorescence intensity of intracellular FITC was quantified using Zen software.

### 2.5 In vitro anti-oxidant and anti-inflammatory effects of Co_3_O_4_@MMT

We evaluated intracellular ROS generation through DCFH-DA staining. Specifically, RAW264.7 cells were seeded into 24-well plates at a density of 1 × 10^5^ cells per well and incubated for 24 h. Following this, the cells underwent LPS or H_2_O_2_ stimulation and were treated with Co_3_O_4_ (10 μg/mL), MMT (40 μg/mL), or Co_3_O_4_@MMT (50 μg/mL) for another 24-h period. After the treatment phase, we incubated the cells with 10 μM DCFH-DA at 37 °C for 30 min. The cells were then fixed using 4% paraformaldehyde for 20 min, followed by a 10-min counterstaining step with DAPI. We captured intracellular fluorescence images using an inverted fluorescence microscope to assess ROS levels and performed semi-quantitative analysis of the fluorescence intensity using ZEN software. The antioxidant activity of Co_3_O_4_@MMT was evaluated by measuring intracellular SOD and GSH-Px levels. RAW264.7 cells were seeded in 35 mm dishes at a density of 1 × 10^7^ cells per dish and cultured for 24 h, followed by LPS stimulation and the aforementioned treatments for an additional 24 h. After treatment, the cells were harvested in 1 mL PBS, lysed by three freeze-thaw cycles between liquid nitrogen and a 37 °C water bath, and centrifuged to obtain the supernatant. Protein concentrations were determined using a BCA protein assay kit. SOD and GSH-Px activities were measured using respective assay kits by mixing supernatant aliquots with detection reagents per the manufacturer's protocols. Absorbance was recorded at 450 nm for SOD and 412 nm for GSH-Px using a microplate reader. Enzyme activities were calculated according to the provided formulas and expressed as SOD (U/mg protein) and GSH-Px (nmol/min/mg protein).

For gene expression analysis, RAW264.7 cells were inoculated in 6-well plates (4 × 10^6^ cells per well) and allowed to grow overnight. Experimental groups consisted of untreated macrophages, LPS-activated cells, and LPS-stimulated macrophages treated with Co_3_O_4_, MMT, or Co_3_O_4_@MMT at the aforementioned concentrations. Following a 24-h treatment, we isolated total RNA using a commercial extraction kit, strictly adhering to the manufacturer's protocols. We determined RNA concentration and quality with a NanoDrop 2000 spectrophotometer. Subsequently, we reverse-transcribed 1.0 μg of the total RNA into cDNA. Finally, we performed quantitative real-time PCR to measure the mRNA expression of TNF-α, IL-1β, IL-6, COX-2, IL-10 and Arg-1 calculating relative gene expression levels via a real-time fluorescence PCR system. The expression levels of key signaling proteins, specifically the total forms of p-STAT1, p-STAT6 and p-p65 in RAW264.7 cells were assess by Western Blot (WB). Briefly, cellular proteins were extracted using RIPA lysis buffer containing protease inhibitors, and their concentrations were measured with a BCA protein assay kit. The samples were subjected to gel electrophoresis, membrane transfer, and blocking, followed by incubation with primary antibodies against p-p65, p-STAT1, and p-STAT6. Protein bands were detected with an enhanced chemiluminescence (ECL) kit and visualized using a gel imaging system. Densitometric analysis was performed using Gel-Pro software, and β-actin was employed as the loading control for normalization.

### 2.6 In vitro intestinal mucosal barrier repair performance of Co_3_O_4_@MMT

To investigate the capacity of Co_3_O_4_@MMT to mitigate oxidative damage and facilitate barrier repair, we established five experimental groups: a Normal group (untreated), an H_2_O_2_ model group (64 μM H_2_O_2_), and three treatment groups receiving 64 μM H_2_O_2_ supplemented with MMT (40 μg/mL), Co_3_O_4_ (10 μg/mL), or Co_3_O_4_@MMT (50 μg/mL).

We first evaluate the effect of Co_3_O_4_@MMT treatment on mitochondrial function in Caco-2 cells, cells were seeded into 24-well plates at a density of 1 × 10^5^ cells per well with the treatments performed as described above. The culture medium was then removed, and the cells were incubated with either 500 μL of JC-1 working solution for 30 min or 500 μL of PBS containing 500 nM Mito-SOX probe for 30 min at 37 °C. After staining, the cells were rinsed with PBS, nuclei were labeled with Hoechst 33,342, and fluorescence imaging was performed using an inverted fluorescence microscope. Quantitative analysis of probe fluorescence was carried out with ZEN software [32].

Then, we first evaluated the cytoprotective effect of the materials against oxidative stress via the CCK-8 assay. On the first day, we seeded cells into 96-well plates at a density of 2 × 10^4^ cells per well and allowed them to adhere overnight. On the second day, we administered the specified treatments to each group. By the third day, we introduced the CCK-8 staining solution and measured the optical density at 450 nm to determine the survival rate of the cells.

To assess cell proliferation and apoptosis, we inoculated cells into 24-well plates at a density of 1 × 10^5^ cells per well on the first day. After an overnight incubation for cell attachment, we performed the relevant assays on the third day following the treatment period. For the EdU experiment, we incubated the cells with a 10 μM EdU solution and Hoechst 33,342. For apoptosis analysis, we utilized the TUNEL staining technique. We then employed an inverted fluorescence microscope to capture images and analyze the cellular response to oxidative challenge.

In the wound healing experiment, we cultured cells in 6-well plates until a confluent monolayer was formed. We then created a linear scratch using a sterile pipette tip and gently rinsed the wells with PBS to remove floating debris. After applying the indicated treatments, we monitored the migration of the cells and recorded the status of the scratch at 0 and 24 h using an inverted microscope. In the FITC-dextran permeability assay, Caco-2 cells were seeded in a 24-well transwell plate and incubated for 10-15 days. The cells were then treated as described above for 24 h, followed by two washes with PBS. FITC-dextran solution (1 mg/mL) was added to the apical chamber, and 2 h of incubation was followed, The medium in the basolateral chamber was collected, and fluorescence was measured using a multifunctional microplate reader at an excitation wavelength of 480 nm and an emission wavelength of 530 nm.

Finally, we utilized RT-PCR to quantify the mRNA expression of key intestinal junction proteins. On the first day, we seeded cells in 6-well plates at a density of 1 × 10^6^ cells per well. We administered the grouped treatments on the second day and harvested total RNA on the third day. We then measured the transcriptional levels of Claudin-1, Occludin, ZO-1, HIF-1α, VEGFA to evaluate the impact of Co_3_O_4_@MMT on the genetic regulation of the intestinal barrier's structural integrity. WB was performed to determine the protein expression levels of HIF-1α, VEGFA, and GLUT1, thereby examining whether Co_3_O_4_@MMT preserves intestinal epithelial barrier integrity via the HIF-1 signaling pathway. The experimental groups were identical to those described above, except that an additional group was treated with H_2_O_2_, Co_3_O_4_@MMT and PX-478 (20 μM). After 24 h of incubation, total cellular proteins were extracted and analyzed by Western blot analysis, with β-actin used as the internal reference for quantitative normalization.

### 2.7 Anti-oxidant, anti-inflammatory, and mucosal barrier repair mechanisms of Co_3_O_4_@MMT

To uncover the antioxidant, anti-inflammatory, and mucosal repair mechanisms of Co_3_O_4_@MMT, we harvested LPS-stimulated macrophages after nanozyme incubation and carried out RNA sequencing. TRIzol reagent was employed for total RNA isolation. A NanoDrop 2000 spectrophotometer was used to evaluate RNA quantity and purity; A260/A280 ratios near 2.0 and A260/A230 values within 2.0-2.2 were regarded as acceptable. RNA integrity was further verified on an Agilent 5300 Bioanalyzer, and specimens with an RNA integrity number (RIN) above 7 proceeded to library preparation. Raw count tables were processed and visualized in the R framework through several packages. Specifically, the limma package normalized the count matrix to guarantee cross-sample consistency. The normalized expression data then underwent principal component analysis (PCA) to evaluate global variance and potential clustering trends. Genes with differential expression were selected according to combined statistical and biological significance cutoffs. Volcano plots were constructed to exhibit the distribution of up-versus down-regulated transcripts, while the pheatmap package rendered heatmaps depicting sample-wise expression profiles via a color-gradient scale. Finally, the clusterProfiler package performed GO and KEGG enrichment analyses on the DEG set, revealing overrepresented biological processes, cellular components, molecular functions, and signaling cascades linked to the observed transcriptional responses.

### 2.8 Evaluation of the therapeutic efficacy of Co_3_O_4_@MMT in DSS-induced colitis mice

To determine the specific binding and coverage of Co_3_O_4_@MMT on damaged intestinal mucosa, female ICR mice were acclimated for 7 days. Experimental colitis was induced in the model group by supplying 5% (w/v) DSS in drinking water for nine consecutive days, whereas age-matched healthy mice were maintained on normal drinking water. Mice in both groups were orally gavaged with FITC-Co_3_O_4_@MMT (200 mg/kg) on days 5, 7, and 9. Following the final gavage on day 9, the distribution of FITC-Co_3_O_4_@MMT was dynamically tracked in healthy and DSS-induced colitis mice at 0, 1, 3, 6, and 9 h using an in vivo imaging system (AniView100). At the 9 h time point, all mice were euthanized, and colon tissues were harvested for ex vivo imaging to further assess the distribution of FITC-Co_3_O_4_@MMT within the colon. The fluorescence intensity of FITC-Co_3_O_4_@MMT was further quantitatively analyzed [33].

To assess the therapeutic efficacy of Co_3_O_4_@MMT against DSS-triggered colitis, female ICR mice underwent a 7-day acclimation phase. Colitis was initiated by supplying 5% (w/v) DSS in drinking water over nine consecutive days. Age-matched healthy controls (n = 5) received normal drinking water throughout. Following DSS exposure, animals were randomly divided into four groups (n = 5 per group) and received oral gavage on days 5, 7, and 9 with vehicle, MMT (160 mg/kg), Co_3_O_4_ (40 mg/kg), or Co_3_O_4_@MMT (200 mg/kg). Body mass, stool form, and fecal blood content were recorded daily to gauge disease progression. Disease activity index (DAI) was computed as previously reported [34], integrating scores for weight reduction (0 = none; 1 = <5%; 2 = 5-10%; 3 = 10-15%; 4 = >15%), stool consistency (0 = formed; 1-2 = soft; 3-4 = liquid), and fecal occult or gross bleeding (0-1 = negative; 2-3 = positive; 4 = visible blood). Starting on day 9, mice were fasted, and on day 11, retro-orbital blood was drawn for hematological assays. At study termination, all subjects were euthanized. Colon lengths were measured, and spleens were weighed to derive spleen indices. For histopathological examination, colonic tissues and vital organs were rinsed, paraffin-embedded, sectioned, and stained with H&E. Histology scores combined subscores (0-3 each) for epithelial disruption, edema, erosion, and leukocyte infiltration, yielding a final range of 0 (intact) to 6 (severe damage with extensive infiltration [35].

To explore in vivo immune modulation and mucosal barrier preservation, colon specimens underwent immunohistochemistry (IHC) and immunofluorescence (IF) staining and Western Blot (WB) per established protocols. After sectioning, tissues were incubated with primary antibodies. IHC targeted TNF-α, IL-1β, and IL-6 to visualize cytokine expression. IF employed antibodies against iNOS and CD206 for macrophage phenotype assessment, alongside antibodies against Claudin-5, Occludin-1, and ZO-1 to evaluate tight junction integrity. Fluorescent signals were captured with an inverted microscope under uniform acquisition parameters for inter-group comparison. To further quantify the expression levels of tight junction proteins, WB was performed to detect the protein expression of Claudin-1, ZO-1 and Occludin in colon tissue homogenates. Fresh feces were harvested separately from each cohort, instantly cryopreserved in liquid nitrogen, and stored at −80 °C until processing. Gut microbial profiles were determined via 16S rRNA amplicon sequencing to examine Co_3_O_4_@MMT-mediated microbiome remodeling in colitis. Total DNA was extracted with the E. Z.N.A. Soil DNA Kit. DNA purity and concentration were verified by 1.0% agarose gel electrophoresis and Nanodrop 2000 readings. The V3-V4 hypervariable region was amplified with specific primers on an ABI GeneAmp 9700 system. Following Majorbio's standard pipe line, sequencing was performed on an Illumina MiSeq platform. Sequences sharing ≥97% identity were clustered into OTUs using UPARSE (v7.1). Representative OTU sequences were taxonomically assigned against the Silva database (confidence threshold ≥0.7). All downstream analyses were executed on the Majorbio Biocloud platform.

### 2.9 Statistical analysis

All values are expressed as mean ± standard deviation (SD). Each data set was derived from at least three independent parallel experiments. For statistical comparison, one-way analysis of variance (ANOVA) was performed using GraphPad Prism 9 (GraphPad Software, USA). A p-value less than 0.05 was considered statistically significant. Significance is indicated as ∗p < 0.05, ∗∗p < 0.01, and ∗∗∗p < 0.001; “ns” signifies no statistically significant difference.

## 3 Results and discussion

### 3.1 Preparation and physicochemical characterization of Co_3_O_4_@MMT nanocomposites

The Co_3_O_4_@MMT nanoplatform was constructed via a controlled exfoliation and in situ growth strategy. We first examined the morphological evolution of the MMT scaffold. As revealed by scanning electron microscopy (SEM) in Fig. S1A, pristine MMT displayed a dense, bulk-like microstructure. Following 48 h of ammonia-assisted mechanical stirring, the interlayer electrostatic forces were sufficiently disrupted, yielding thin and well-defined MMT nanosheets (Fig. S1B).

To identify the optimal composition for therapeutic application, we systematically compared three mass ratios of Co_3_O_4_@MMT (1:9, 2:8, and 3:7) in terms of hydrodynamic diameter, polydispersity index (PDI) (Fig. S2 and Table S1), and surface charge (Fig. S3). The 3:7 formulation exhibited a marked increase in both particle size (1406.0 nm and PDI 0.40), indicative of pronounced aggregation. Such aggregation is undesirable, as it typically reduces the exposure of catalytically active sites and compromises colloidal stability [36]. In contrast, the 3:7 formulation exhibited a positive zeta potential (32.60 mV), whereas the 1:9 and 2:8 groups retained negative surface charges of −16.73 mV and −11.02 mV, respectively. Given that the inflamed colonic epithelium is characterized by overexpression of positively charged proteins [37], a strongly negative surface charge is advantageous for facilitating electrostatic adhesion and prolonging mucosal retention. On this basis, the 3:7 ratio was excluded from further consideration. Furthermore, the catalytic activities of Co_3_O_4_@MMT at mass ratios of 1:9 and 2:8 were investigated, revealing that both formulations exhibited comparable catalytic performance (Figs. S4 and S5). And the final selection between the 1:9 and 2:8 ratios was guided by a hemolysis assay assessing blood compatibility (Figs. S6 and S7). Notably, a higher MMT proportion correlated with increased erythrocyte lysis; the 1:9 group caused evident hemolysis (17.17%), whereas the 2:8 group exhibited minimal hemolytic activity (1.33%), well below the clinically safe threshold of 5%. This observation aligns with previous reports that excessive silicate content may perturb cell membranes [38]. Considering the trade-off among structural uniformity, surface charge-mediated targeting, and biosafety, the 2:8 ratio was therefore selected as the optimized formulation for all subsequent experiments.

**Fig. 1:**
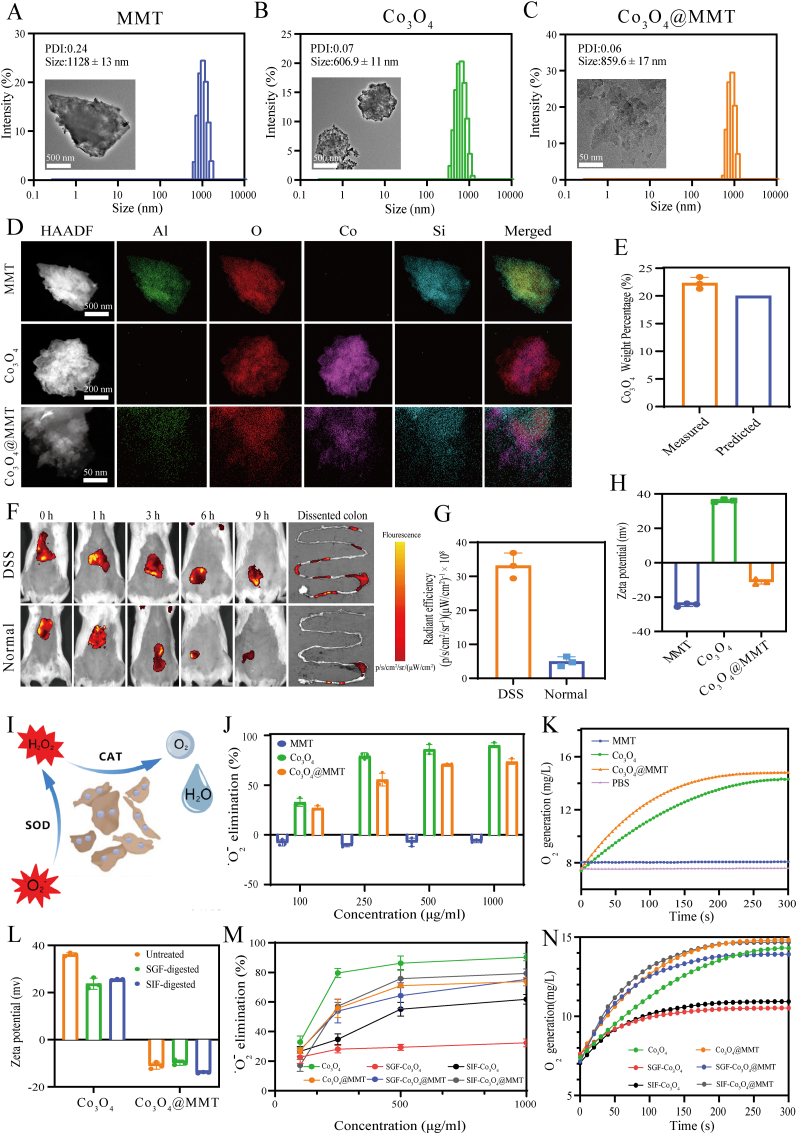
(A-C) TEM images and corresponding size distribution of MMT nanosheet, Co_3_O_4_ and Co_3_O_4_@MMT. (D) HAADF image and corresponding EDS elemental mapping, showing uniform distribution of Al, O, Co and Si. (E) Co_3_O_4_ weight percentage in Co_3_O_4_@MMT predicted or measured by ICP-OES. (F, G) Time-dependent biodistribution of FITC-Co_3_O_4_@MMT in the colon after rectal administration. (H) Zeta potential of MMT, Co_3_O_4_ and Co_3_O_4_@MMT (I) Schematic illustration of the sequential SOD- and CAT-like catalytic cascade for ROS scavenging. (J, K) SOD-mimetic and CAT-mimetic activities of Co_3_O_4_@MMT. (L) Zeta potential changes of Co_3_O_4_ and Co_3_O_4_@MMT after exposure to simulated gastric fluid (SGF) and simulated intestinal fluid (SIF). (M, N) Retention of SOD- and CAT-like activities after gastrointestinal digestion.

Comprehensive characterization was then performed on the optimized Co_3_O_4_@MMT (2:8) nanocomposite to verify its hybrid architecture. Transmission electron microscopy (TEM) and corresponding size distribution analysis (Fig. 1A–C) first confirmed the successful decoration of Co_3_O_4_ nanoparticles onto the exfoliated MMT sheets. To provide orthogonal evidence of this interfacial assembly, a panel of complementary techniques was employed. SEM imaging revealed a distinctly roughened surface topography following Co_3_O_4_ deposition (Fig. S8), while Fourier transform infrared (FTIR) spectroscopy was employed to characterize the chemical structures of MMT, Co_3_O_4_, and Co_3_O_4_@MMT. The spectrum of Co_3_O_4_@MMT retained the characteristic absorption peaks of both MMT (Si-O-Si, Si-O-Al) and Co_3_O_4_ (Co-O stretching at 665 cm^−1^), confirming the successful integration of Co_3_O_4_ nanoparticles onto the MMT nanosheets (Fig. S9). X-ray photoelectron spectroscopy (XPS) further confirmed the surface chemical states, showing Co 2p_3/2_ and Co 2p_1/2_ peaks consistent with the mixed Co^2+^/Co^3+^ oxidation states typical of spinel Co_3_O_4_ (Fig. S10). The XRD pattern of Co_3_O_4_@MMT exhibited the characteristic peaks of both MMT and Co_3_O_4_, confirming the successful loading of Co_3_O_4_ onto the MMT matrix without altering their original crystalline structures (Fig. S11). The Raman spectrum of Co_3_O_4_@MMT exhibited the characteristic vibrational bands of both MMT and Co_3_O_4_, further confirming the successful integration of Co_3_O_4_ with the MMT matrix (Fig. S12). This multilayered evidence was substantiated by high-angle annular dark-field (HAADF) imaging and energy-dispersive X-ray spectroscopy (EDS) elemental mapping (Fig. 1D), which demonstrated precise spatial colocalization of the signature elements of MMT (Al, Si) and Co_3_O_4_ (Co). Additionally, inductively coupled plasma (ICP) analysis verified that the actual Co_3_O_4_ loading content (22.3 wt%) closely matched the theoretical design (20 wt%, Fig. 1E), underscoring the reproducibility of the synthesis.

To investigate the selective adhesion of Co_3_O_4_@MMT to the inflamed intestinal surface, we performed in vivo imaging experiments. As shown in Fig. 1F and G, Co_3_O_4_@MMT exhibited markedly prolonged retention in the intestinal tract of DSS-induced colitis mice compared with healthy controls. Strong fluorescence signals were continuously observed in the colitic group after administration, whereas the signals in the normal group rapidly diminished, indicating that Co_3_O_4_@MMT selectively adheres to the surface of inflamed colonic lesions, thereby achieving inflammation-targeted retention. Furthermore, zeta potential measurements confirmed that Co_3_O_4_@MMT retained a negative surface charge (−11.02 mV, Fig. 1H), a property essential for its binding to the positively charged damaged mucosa.

Collectively, these results establish the precise, stable, and scalable fabrication of the Co_3_O_4_@MMT nanozyme platform, providing a reliable foundation for subsequent biological evaluation.

### 3.2 Evaluation of catalytic antioxidant performance and gastrointestinal stability

The multienzymatic antioxidant mechanism of the Co_3_O_4_@MMT nanocomposite was systematically interrogated to delineate its capacity for mitigating oxidative stress. As schematically depicted in Fig. 1I, the material operates through a tandem catalytic cascade. It first mimics superoxide dismutase (SOD) to disproportionate superoxide anions (·O_2_^−^) into H_2_O_2_, followed by catalase (CAT)-like activity that decomposes H_2_O_2_ into innocuous water and molecular oxygen [39]. To quantify these mimetic functions, we measured the superoxide anion scavenging rate, which confirmed that Co_3_O_4_@MMT possesses robust SOD-like efficiency (73.81%, Fig. 1J). The CAT-like performance was further substantiated by real-time monitoring of oxygen generation during H_2_O_2_ decomposition (Fig. 1K), demonstrating the effective catalytic conversion capacity of the platform. Additionally, as shown in Fig. S13, the Michaelis-Menten kinetic analysis revealed that Co_3_O_4_@MMT exhibited a slightly higher maximum reaction velocity (V_max_ = 62.96 mg/L/min) than Co_3_O_4_ alone (V_max_ = 49.03 mg/L/min), along with an increased K_m_ value (from 331.03 to 478.15 mM). Moreover, given that ·OH represent the most reactive and deleterious reactive species toward cellular biomolecules [40], we evaluated the ·OH scavenging capability of the nanocomposite. The results indicated that Co_3_O_4_@MMT maintains excellent •OH quenching activity (99.15%, Fig. S14), thereby furnishing a comprehensive enzymatic defense arsenal against diverse oxidative insults.

Beyond catalytic potency, the structural and functional resilience of the nanozyme within the gastrointestinal tract constitutes a critical prerequisite for efficacious oral delivery [41]. To recapitulate these physiological barriers, the materials were pre-incubated in simulated gastric fluid (SGF) and simulated intestinal fluid (SIF) prior to re-evaluation of their physicochemical and catalytic properties. As shown in Fig. 1L, the zeta potential of pristine Co_3_O_4_ nanoparticles underwent pronounced fluctuations after sequential SGF/SIF exposure, indicative of surface corrosion or colloidal destabilization under harsh gastrointestinal conditions. In sharp contrast, the zeta potential of Co_3_O_4_@MMT remained remarkably stable, suggesting that the MMT nanosheets serve as a protective buffer that preserves the interfacial characteristics of the composite. This shielding effect is likely attributable to the high aspect ratio and negative surface charge of exfoliated MMT, which may provide a physical barrier against acid and enzymatic attack [30].

The impact of gastrointestinal transit on catalytic performance was further evaluated by reassessing the enzyme-like activities of the materials post-digestion (Fig. 1M and N, and S15). Whereas bare Co_3_O_4_ nanoparticles exhibited a marked decline in both SOD-like and CAT-like activities following SGF/SIF treatment, the Co_3_O_4_@MMT nanocomposite retained nearly all of its original multienzymatic functionality. These findings demonstrate that the MMT scaffold not only provides physical isolation for the embedded Co_3_O_4_ active sites but also preserves their redox integrity against denaturation by gastric acid and proteolytic enzymes. Such enhanced gastrointestinal stability establishes a robust experimental foundation for the subsequent application of Co_3_O_4_@MMT as an orally administrable nanotherapeutic for inflammatory bowel disease, wherein sustained antioxidant capacity within the inflamed colonic milieu is paramount.

### 3.3 Antioxidant and anti-inflammatory effects and mechanisms of Co_3_O_4_@MMT

**Fig. 2:**
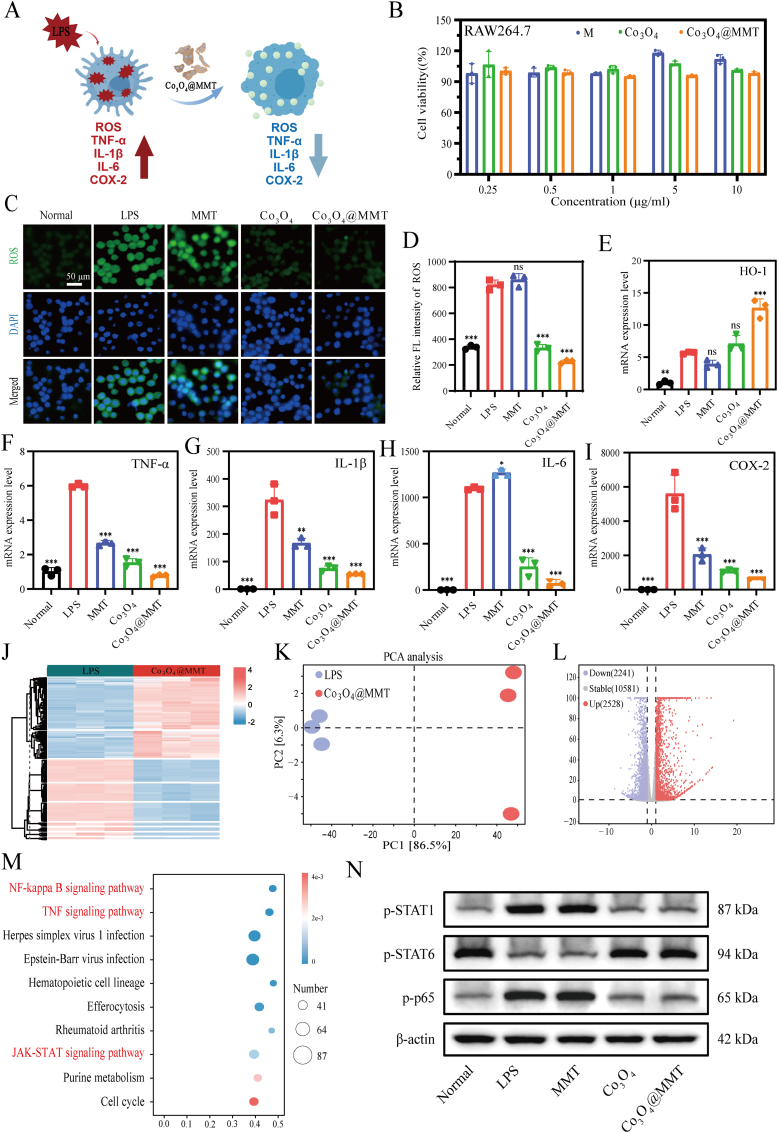
(A) Schematic of the ROS–inflammation loop in LPS-stimulated macrophages. (B) Cell viability assessed by CCK-8 assay. (C, D) Intracellular ROS levels detected by DCFH-DA staining and semi-quantitative analysis. (E) mRNA expression of the antioxidant gene HO-1. (F–I) mRNA levels of pro-inflammatory markers (TNF-α, IL-1β, IL-6, COX-2). (J) Heatmap of differentially expressed genes from RNA-seq. (K) PCA plot showing global transcriptome shifts. (L) Volcano plot of DEGs between LPS and Co_3_O_4_@MMT groups. (M) KEGG enrichment analyses of DEGs (N)Protein expression levels of p-STAT1, p-STAT6, and p-p65 in RAW 264.7 cells. All statistical analyses were compared by comparing the LPS group with other groups (n = 3), with ∗p < 0.05, ∗∗p < 0.01, ∗∗∗p < 0.001, and ns indicating no significant difference.

Fig. 2A delineates the proposed mechanism of Co_3_O_4_@MMT within the inflammatory cascade. Upon lipopolysaccharide (LPS) stimulation, RAW264.7 macrophages undergo robust generation of ROS and pro-inflammatory mediators, establishing a self-perpetuating cycle between oxidative injury and inflammatory signaling [42]. By efficiently scavenging intracellular ROS, Co_3_O_4_@MMT disrupts this deleterious feedback loop, thereby conferring potent anti-inflammatory effects. This intervention not only alleviates oxidative burden but also curtails downstream inflammatory amplification—a dual benefit that distinguishes multifunctional nanozymes from conventional single-target therapeutics.

Prior to functional evaluation, the biocompatibility of each formulation was examined using the CCK-8 assay. At a normalized Co_3_O_4_ concentration of 10 μg/mL (equivalent to 40 μg/mL MMT, 10 μg/mL Co_3_O_4_, and 50 μg/mL Co_3_O_4_@MMT), none of the treatments elicited appreciable cytotoxicity (Fig. 2B). Live/dead fluorescent staining further corroborated the excellent cytocompatibility of this working concentration (Fig. S16). Accordingly, this dosage was adopted for all subsequent in vitro experiments. Additionally, as shown in Fig. S17, efficient cellular uptake of FITC-labeled Co_3_O_4_@MMT by RAW 264.7 cells was observed, as evidenced by the strong green fluorescence signal localized within the cells in the merged images.

We next assessed the ROS scavenging aptitude of the nanocomposite via DCFH-DA staining. As presented in Fig. 2C, cells treated with Co_3_O_4_@MMT exhibited markedly attenuated DCFH fluorescence relative to the LPS-stimulated control; semi-quantitative analysis confirmed a significant reduction in fluorescence intensity (Fig. 2D). Furthermore, a CCK-8 assay was first performed to determine the appropriate H_2_O_2_ concentration for inducing oxidative stress in RAW 264.7 cells. As shown in Fig. S18, treatment with 200 μM H_2_O_2_ reduced cell viability to approximately 50% of the control level. Therefore, this concentration was selected for subsequent oxidative stress modeling. After H_2_O_2_ stimulation, intracellular ROS levels were assessed using the DCFH-DA probe (Fig. S19). Compared with the oxidative stress group (H_2_O_2_ alone), the fluorescence intensity was markedly decreased in the Co_3_O_4_@MMT-treated group, indicating that Co_3_O_4_@MMT effectively alleviated H_2_O_2_-induced oxidative stress in RAW 264.7 cells. We also measured the activities of intracellular antioxidant enzymes (SOD and GSH-Px) in Fig. S20. Following Co_3_O_4_@MMT treatment, these enzyme activities were significantly increased, indicating that the material can regulate the cellular antioxidant defense system. Moreover, reverse transcription PCR (RT-PCR) revealed that Co_3_O_4_@MMT significantly upregulated the transcriptional level of heme oxygenase-1 (HO-1, Fig. 2E), a pivotal antioxidant enzyme that catabolizes heme into biliverdin, ferrous iron, and carbon monoxide—effector molecules with established cytoprotective and immunomodulatory properties [43]. This elevation implies that Co_3_O_4_@MMT may augment endogenous antioxidant defense machinery in parallel with its direct ROS-scavenging activity.

To further probe its anti-inflammatory potency, we quantified the mRNA expression of canonical pro-inflammatory cytokines. Compared with the LPS group, Co_3_O_4_@MMT treatment robustly suppressed the transcript levels of TNF-α (Fig. 2F), IL-1β (Fig. 2G), IL-6 (Fig. 2H), and COX-2 (Fig. 2I). Additionally, we measured the levels of anti-inflammatory cytokines (IL-10 and Arg-1) in Fig. S21. Compared with the LPS group, the Co_3_O_4_@MMT-treated group exhibited significantly increased levels of these anti-inflammatory factors. These results collectively demonstrate that Co_3_O_4_@MMT intervenes at the transcriptional level to dampen the production of key inflammatory mediators, thereby attenuating the acute inflammatory response.

For a more comprehensive mechanistic interrogation, we performed RNA sequencing (RNA-seq) on LPS-activated macrophages with or without Co_3_O_4_@MMT treatment. The unsupervised hierarchical clustering heatmap (Fig. 2J) and principal component analysis (PCA, Fig. 2K) both revealed a pronounced separation of the transcriptional profiles between the LPS and Co_3_O_4_@MMT groups, underscoring the profound impact of the nanocomposite on the macrophage transcriptome. The volcano plot (Fig. 2L) identified a total of 4769 differentially expressed genes (DEGs), with 2528 upregulated and 2241 downregulated in response to Co_3_O_4_@MMT (|log_2_FC| ≥ 1, adjusted p < 0.05). Subsequent Kyoto Encyclopedia of Genes and Genomes (KEGG) pathway enrichment analysis disclosed that Co_3_O_4_@MMT significantly modulated several classic inflammatory signaling cascades, including the NF-κB [44], TNF [45], and JAK-STAT [46] pathways (Fig. 2M). The coordinated suppression of these axes corroborates the nanocomposite's capacity to dismantle the interconnected ROS-inflammation network. In Fig. 2N and S22, to further explore the anti-inflammatory mechanism of the material, we first measured the levels of p-STAT1 and p-STAT6 by western blotting. Compared with the LPS group, the Co_3_O_4_@MMT treatment group showed decreased p-STAT1 and increased p-STAT6 expression, supporting our hypothesis that the material exerts anti-inflammatory effects by modulating the JAK-STAT signaling pathway. In addition, we measured the level of p-p65, a key protein in the NF-κB pathway. Co_3_O_4_@MMT treatment significantly downregulated p-p65 expression, suggesting that the material may also suppress inflammation by blocking p65 receptor-mediated signaling. The additional Gene Ontology (GO) analysis further revealed significant enrichment in biological processes related to nitrogen compound metabolism and organic substance metabolism (Fig. S23). This metabolic reprogramming suggests that Co_3_O_4_@MMT may rewire the immunometabolic state of activated macrophages—shifting them away from a pro-inflammatory glycolytic phenotype toward a quiescent state—thereby reducing the biosynthesis of inflammatory mediators. Collectively, these transcriptomic insights establish that Co_3_O_4_@MMT orchestrates a multifaceted anti-inflammatory response through concurrent ROS elimination, antioxidant enzyme activation, inflammatory pathway blockade, and immunometabolic regulation.

### 3.4 Assessment of in vitro mucosal barrier repair and underlying mechanisms

To evaluate the reparative effects of Co_3_O_4_@MMT on the intestinal epithelial barrier, we first confirmed its biosafety in Caco-2 cells. CCK-8 viability assays and live/dead fluorescent staining revealed no significant cytotoxicity at a normalized Co_3_O_4_ concentration of 10 μg/mL (Figs. S24 and S25), establishing a safe and reliable working dose for subsequent functional investigations. Additionally, as shown in Fig. S26, efficient cellular uptake of FITC-labeled Co_3_O_4_@MMT by Caco-2 cells was observed, as evidenced by the strong green fluorescence signal localized within the cells in the merged images. An oxidative injury model was then established by exposing Caco-2 monolayers to 64 μM H_2_O_2_ for 24 h, which reduced cell viability to approximately 40% of the untreated control (Fig. S27)—a level of oxidative stress commonly employed to recapitulate the harsh redox microenvironment characteristic of active ulcerative colitis.

**Fig. 3:**
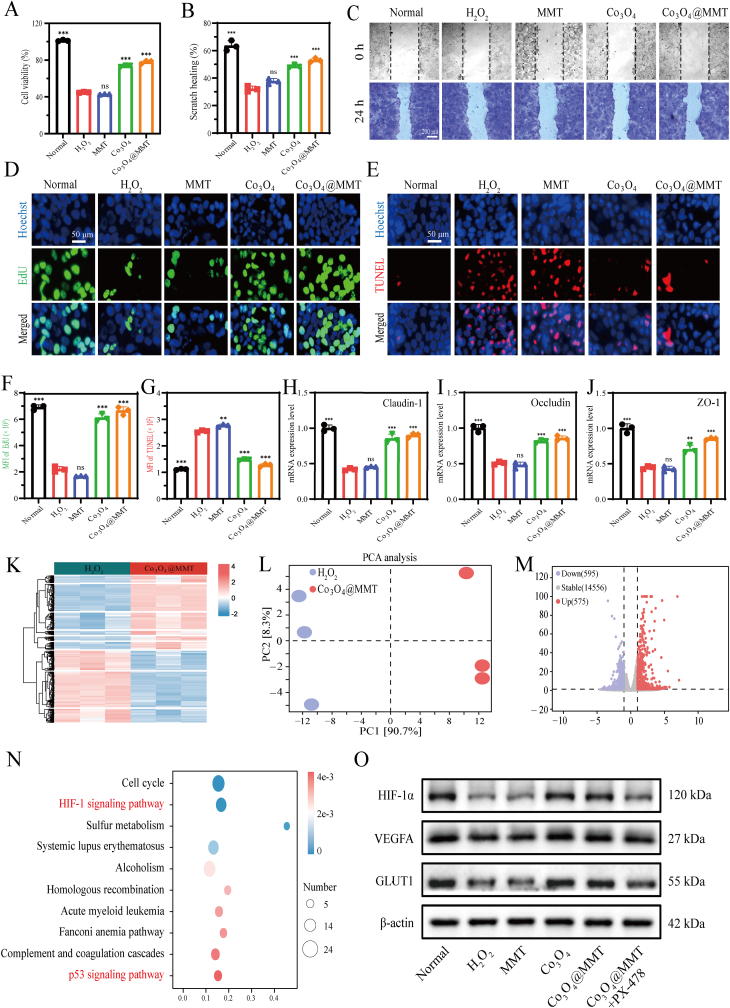
(A)CCK-8 assay demonstrating that the material alleviates oxidative stress and enhances cell survival. (B)Quantitative analysis of scratch healing rate. (C) Scratch wound healing assay showing cell proliferation capability. (D, E) EdU staining and PI staining assays of Caco-2 cells following various treatments. (F, G) Quantitative analysis of EdU-positive cells, TUNEL-positive cells, and scratch closure rate. (H–J) mRNA expression levels of tight-junction proteins (Claudin-1, Occludin and ZO-1) in Caco-2 cells measured by quantitative qRT-PCR. (K) Heatmap of differentially expressed genes from RNA-seq. (L) PCA plot showing global transcriptome shifts. (M) Volcano plot of DEGs between H_2_O_2_ and Co_3_O_4_@MMT groups. (N) KEGG enrichment analyses of DEGs. (O) Protein expression levels of HIF-1α, VEGFA and GLUT1 in Caco-2 cells. All statistical analyses were performed by comparing the H_2_O_2_ group with other groups (n = 3), with ∗p < 0.05, ∗∗p < 0.01, ∗∗∗p < 0.001, and ns indicating no significant difference.

Under these pathological conditions, we assessed the barrier-protective capacity of Co_3_O_4_@MMT. As demonstrated in Fig. 3A, treatment with Co_3_O_4_@MMT significantly attenuated H_2_O_2_-induced oxidative stress injury and restored cellular viability to near-normal levels. Scratch wound healing assays demonstrated that H_2_O_2_ stimulation markedly impaired epithelial migration, whereas Co_3_O_4_@MMT treatment restored wound closure rates to near-normal levels (53.09% vs. 63.49% at 24 h, Fig. 3B and C). This pro-migratory effect was further supported by EdU incorporation assays, which showed that Co_3_O_4_@MMT preserved proliferative activity compared with the H_2_O_2_-alone group (Fig. 3D–F). Concurrently, TUNEL staining revealed that Co_3_O_4_@MMT significantly attenuated H_2_O_2_-induced apoptosis (Fig. 3E–G). Additionally, as shown in Fig. S28, the FITC-dextran permeability assay was performed on Caco-2 cell monolayers. Compared with the H_2_O_2_ group, Co_3_O_4_@MMT treatment significantly reduced the fluorescence intensity of FITC-dextran in the receiver chamber, indicating that the material effectively protected intestinal epithelial barrier integrity. Then JC-1 and Mito-SOX staining assays were performed to evaluate mitochondrial function in Caco-2 cells (Figs. S29 and S30). Compared with the H_2_O_2_ group, Co_3_O_4_@MMT treatment restored the mitochondrial membrane potential (increased JC-1 aggregates) and significantly reduced mitochondrial ROS levels (decreased Mito-SOX fluorescence), indicating that the material protects intestinal epithelial cells from oxidative damage.

At the molecular level, RT-PCR analysis indicated that H_2_O_2_ exposure led to a pronounced downregulation of tight junction proteins Claudin-1, Occludin, and ZO-1 at the mRNA level—an alteration frequently associated with increased intestinal permeability in inflammatory bowel disease. Notably, Co_3_O_4_@MMT treatment effectively reversed this suppression, restoring their transcript levels to values comparable with those of untreated controls (Fig. 3H–J). Collectively, these results demonstrate that Co_3_O_4_@MMT not only protects intestinal epithelial cells from oxidative insult but also actively promotes their recovery by enhancing migration, proliferation, and the expression of junctional complexes.

To elucidate the transcriptional programs underlying this reparative phenotype, we performed RNA-seq on Caco-2 cells subjected to H_2_O_2_ with or without Co_3_O_4_@MMT treatment. Unsupervised hierarchical clustering (Fig. 3K) and principal component analysis (Fig. 3L) revealed a clear separation between the H_2_O_2_ and Co_3_O_4_@MMT groups, indicating that the nanocomposite broadly counteracts oxidative stress-induced transcriptomic dysregulation. The volcano plot (Fig. 3M) identified 1170 differentially expressed genes (|log_2_FC| ≥ 1, adjusted p < 0.05), with 575 upregulated and 595 downregulated following Co_3_O_4_@MMT intervention. KEGG enrichment analysis revealed significant upregulation of several pathways intimately linked to epithelial repair and mucosal regeneration, including HIF-1 signaling [47] and p53 signaling [48](Fig. 3N). Activation of the HIF-1 pathway is particularly noteworthy, given its established role in orchestrating metabolic adaptation, angiogenesis, and barrier preservation during intestinal wound healing. To validate the transcriptomic finding that Co_3_O_4_@MMT activates the HIF-1 pathway, we first measured the levels of HIF-1α and VEGFA by qPCR (Fig. S31). The results showed that both factors were upregulated after material treatment. Furthermore, as shown in Fig. 3O and S32, the protein expression levels of HIF-1α, VEGFA, and GLUT1, three key proteins in the HIF-1 pathway, were all increased following Co_3_O_4_@MMT treatment. Notably, when the specific HIF-1 pathway inhibitor PX-478 was added together with Co_3_O_4_@MMT, the expression of these three proteins was markedly reduced. These results confirm our initial transcriptomic finding that the material activates the HIF-1 signaling pathway in Caco-2 cells. Additionally, Co_3_O_4_@MMT upregulated HIF-1α while downregulating p65 NF-κB. Given the known crosstalk between these two pathways during epithelial repair, it is plausible that their reciprocal regulation contributes to the restoration of intestinal epithelial integrity. GO analysis further demonstrated pronounced enrichment in biological processes associated with mitotic cell cycle progression, DNA replication, and cellular response to stress (Fig. S33), reinforcing the concept that Co_3_O_4_@MMT actively engages proliferative and pro-survival transcriptional programs rather than merely alleviating oxidative injury.

Taken together, these integrated phenotypic and transcriptomic findings establish that Co_3_O_4_@MMT exerts a dual mechanism on the intestinal epithelium: it directly scavenges cytotoxic ROS to mitigate oxidative damage, while simultaneously initiating a transcriptionally orchestrated regenerative response that enhances epithelial proliferation, suppresses apoptosis, and restores tight junction integrity. This coordinated barrier-repair capacity distinguishes Co_3_O_4_@MMT from conventional antioxidants and positions it as a promising orally administrable nanotherapeutic for inflammatory bowel disease.

### 3.5 Therapeutic efficacy of orally administered nanozymes in murine colitis models

**Fig. 4:**
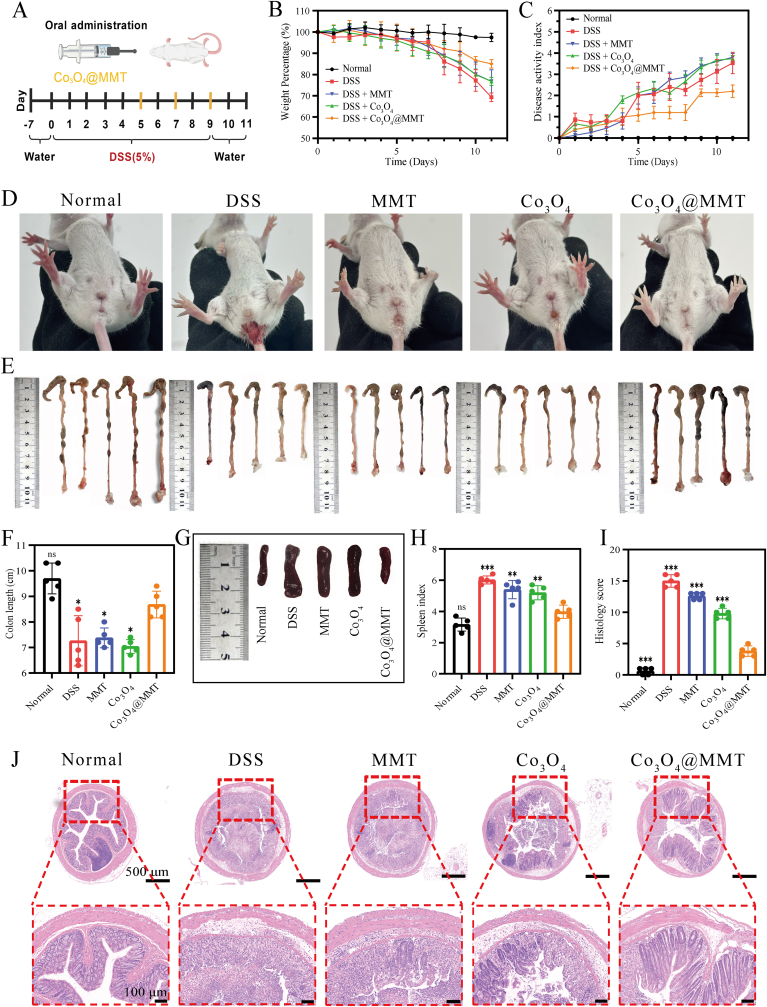
(A) Experimental timeline. (B-D) Body weight change, disease activity index and occurrence of occult blood in UC mice under various treatments on day 11. (E, F) Colon length of mice following the indicated treatments at the end of the study. (G, H) Spleen index and representative images. (I, J) H&E-stained colon sections and histological scoring. Statistical analyses were performed by comparing the Co_3_O_4_@MMT group with other groups (n = 5), with ∗p < 0.05, ∗∗p < 0.01, ∗∗∗p < 0.001, and ns indicating no significant difference.

To interrogate the in vivo therapeutic efficacy of the engineered nanozyme, we established an acute colitis model through administration of dextran sulfate sodium (DSS) in drinking water, with the full experimental timeline delineated in Fig. 4A. Oral gavage of the Co_3_O_4_@MMT nanocomposite prominently alleviated the cardinal clinical hallmarks of experimental colitis. As illustrated in Fig. 4B–D, mice receiving Co_3_O_4_@MMT exhibited markedly slower body weight decline (14.95% loss on day 11 vs. 30.67% in the DSS group), consistently lower Disease Activity Index (DAI) scores (2.2 on day 11 vs. 3.536 in the DSS group), and substantially diminished visible rectal bleeding compared with animals treated with vehicle, MMT, or Co_3_O_4_ alone. These clinical improvements suggest that Co_3_O_4_@MMT effectively interrupts the pro-inflammatory cascade at an early stage, thereby preserving systemic homeostasis.

The therapeutic benefit was further substantiated by macroscopic and histopathological evaluation. DSS-induced colonic shortening—a reliable surrogate for transmural inflammation and tissue fibrosis—was significantly attenuated in the Co_3_O_4_@MMT group (colon length: 8.68 cm vs. 7.26 cm in DSS controls, Fig. 4E and F). Concomitantly, the pronounced splenomegaly observed in diseased animals was markedly suppressed by Co_3_O_4_@MMT treatment, with the spleen index approaching that of healthy controls (Fig. 4G and H). Given that splenic enlargement in colitis models reflects systemic immune activation and extramedullary hematopoiesis driven by chronic inflammation, its reversal indicates that Co_3_O_4_@MMT exerts not only local but also systemic immunomodulatory effects. Histological examination of colonic specimens using H&E staining revealed that Co_3_O_4_@MMT preserved near-normal mucosal architecture, characterized by intact crypt structures, minimal immune cell infiltration, and well-differentiated goblet cells (Fig. 4I). Quantification of histological damage confirmed a significantly reduced composite score in the Co_3_O_4_@MMT group (3.8 vs. 15 in the DSS group), which was statistically indistinguishable from that of healthy mice (Fig. 4J). This architectural preservation is critical, as crypt loss and epithelial erosion are direct drivers of intestinal permeability and antigen translocation in inflammatory bowel disease.

In parallel, we conducted a comprehensive biosafety assessment to support the translational potential of the nanoplatform. Histological sections of major organs—including heart, liver, spleen, lung, and kidney—revealed no discernible morphological abnormalities or lesions across any treatment group (Fig. S34), indicating the absence of overt organ toxicity. Hematological profiling showed that while DSS, MMT, and Co_3_O_4_ groups exhibited a pronounced elevation in white blood cell (WBC) counts, a hallmark of systemic inflammatory response, the Co_3_O_4_@MMT group maintained WBC levels within the normal range, comparable to those of healthy controls (Fig. S35). Other key hematological parameters, including red blood cell (RBC) counts and hemoglobin (HGB) concentration, remained stable across all cohorts, further confirming the hematocompatibility of the material. Moreover, serum biochemical markers of hepatic function—alkaline phosphatase (ALP), aspartate aminotransferase (AST), and alanine aminotransferase (ALT)—were uniformly within physiological reference intervals (Fig. S36), demonstrating that repeated oral administration of Co_3_O_4_@MMT does not induce hepatotoxicity. Collectively, these efficacy and safety data establish that the Co_3_O_4_@MMT nanocomposite combines potent anti-colitic activity with excellent in vivo biocompatibility, positioning it as a promising orally deliverable therapeutic candidate for inflammatory bowel disease. Furthermore, a separate 28-day toxicity study was performed [49]. As shown in Fig. S37, the distribution of Co ions in major organs (heart, liver, spleen, lung, and kidney) was measured after 28 days of treatment. No significant differences in Co ion levels were observed between the Co_3_O_4_@MMT-treated group and the normal control group, indicating that Co_3_O_4_@MMT does not lead to significant accumulation of cobalt in vital organs and exhibits good biosafety.

### 3.6 Targeted remodeling of colonic immune homeostasis and physical barrier repair

**Fig. 5:**
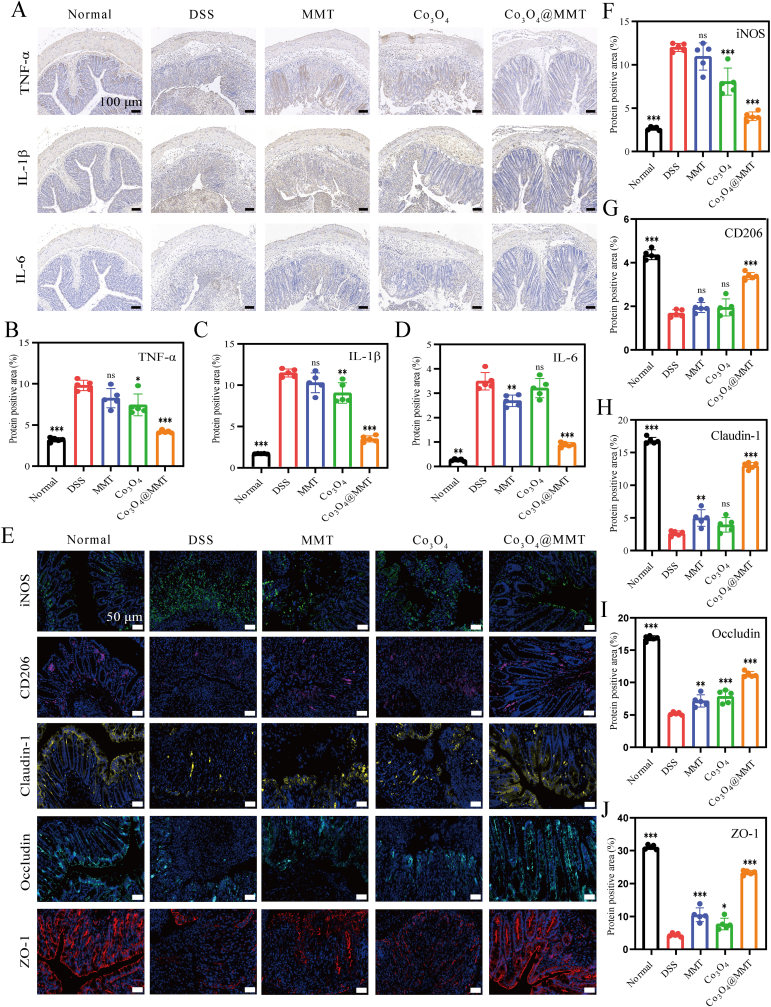
(A) Representative IHC staining of TNF-α, IL-1β, and IL-6 in colon tissues. (B-D) Semi-quantitative analysis of cytokine-positive areas. (E) Immunofluorescence staining of cytokine (iNOS, CD206) and tight-junction proteins (Claudin-1, Occludin and ZO-1). (F, G)Semi-quantification of cytokine iNOS and CD206 expression. (H-J) Semi-quantification of tight-junction proteins Claudin-1, Occludin, and ZO-1. Statistical analyses were performed by comparing the DSS group with other groups (n = 5), with ∗p < 0.05, ∗∗p < 0.01, ∗∗∗p < 0.001, and ns indicating no significant difference.

To further define the molecular basis underlying the therapeutic efficacy of Co_3_O_4_@MMT, we examined its regulatory effects on colonic inflammation and epithelial barrier integrity using immunohistochemical and immunofluorescence approaches. Immunohistochemical staining of colonic sections revealed that Co_3_O_4_@MMT treatment profoundly suppressed the excessive production of pro-inflammatory cytokines. While robust immunoreactivity for TNF-α, IL-1β, and IL-6 was observed in the DSS control group, this signal was markedly diminished in the Co_3_O_4_@MMT cohort (Fig. 5A). Corresponding semi-quantification of the protein-positive area confirmed statistically significant reductions in TNF-α (Fig. 5B), IL-1β (Fig. 5C), and IL-6 (Fig. 5D), indicating effective interruption of the mucosal inflammatory cascade at the protein level.

We next explored whether this anti-inflammatory action was accompanied by reprogramming of the local macrophage compartment. Immunofluorescence staining (Fig. 5E) demonstrated that Co_3_O_4_@MMT potently suppressed the expression of iNOS, a canonical marker of pro-inflammatory M1 macrophages (Fig. 5F). In parallel, the abundance of CD206, a hallmark of the anti-inflammatory and tissue-reparative M2 phenotype, was substantially elevated following treatment (Fig. 5G). This shift from a predominant M1 to an M2-skewed profile reflects a fundamental rebalancing of the mucosal immune microenvironment—a process increasingly recognized as essential for resolving chronic inflammation and initiating tissue repair.

Concurrently, Co_3_O_4_@MMT exhibited a pronounced capacity to restore the integrity of the compromised intestinal epithelial barrier. Immunofluorescence analysis of tight junction complexes revealed that the severe loss of Claudin-5, Occludin-1, and ZO-1 elicited by DSS challenge was effectively reversed upon Co_3_O_4_@MMT administration. Quantitative assessment confirmed that the fluorescence intensities of Claudin-5 (Fig. 5H), Occludin-1 (Fig. 5I), and ZO-1 (Fig. 5J) in the treatment group were restored to levels indistinguishable from those of healthy controls. Furthermore, we evaluated the expression of tight junction proteins (Claudin-1, Occludin, and ZO-1) by western blotting (Fig. S38). Compared with the DSS group, Co_3_O_4_@MMT treatment significantly upregulated the protein levels of Claudin-1, Occludin and ZO-1. These results indicate that Co_3_O_4_@MMT partially restores intestinal barrier integrity by enhancing the expression of specific tight junction proteins. Given that disruption of these junctional proteins directly contributes to increased intestinal permeability and sustained antigen translocation in inflammatory bowel disease, their recovery provides compelling evidence that Co_3_O_4_@MMT actively reconstructs the mucosal barrier rather than merely halting its degradation. Collectively, these findings establish that Co_3_O_4_@MMT orchestrates a coordinated dual-axis repair program—simultaneously resolving inflammation through macrophage repolarization and fortifying epithelial integrity via tight junction restoration.

### 3.7 Modulation of gut microbiota and intestinal microenvironment

**Fig. 6:**
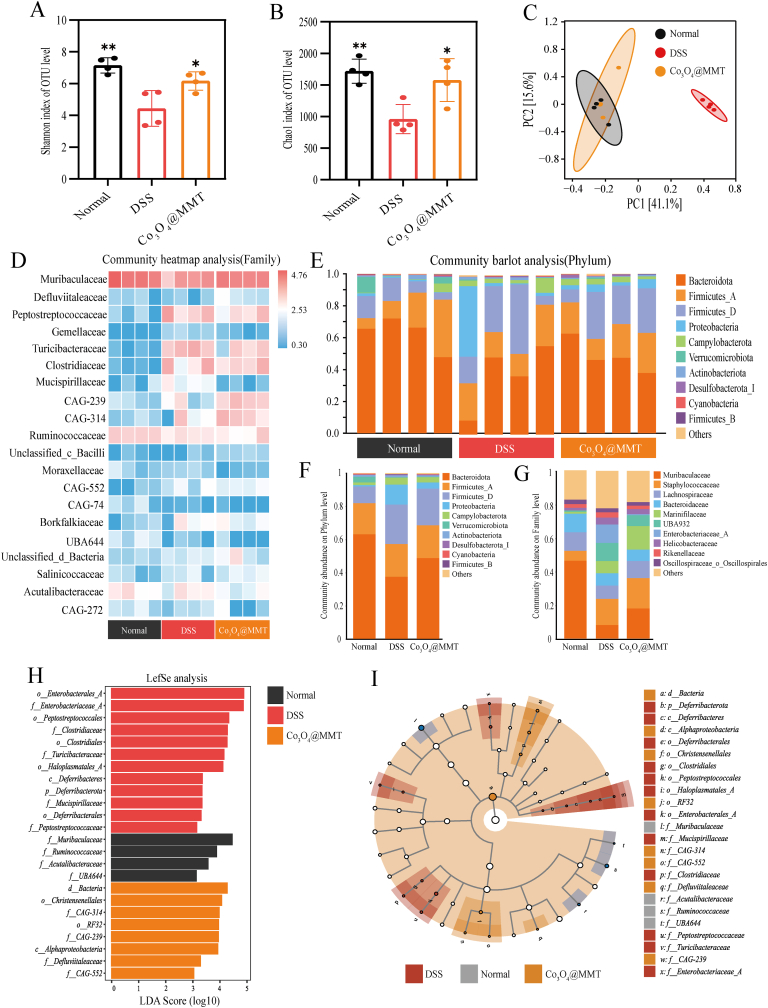
(A, B) α-diversity analysis using the Shannon and Chao1 indices (n = 4). (C) PCoA plot of β-diversity. (D) Relative abundance of microbial clades across different groups. (E-G) Community bar plots depicting the microbial composition. (H, I) LEfSe identifying discriminative bacterial clades enriched in the Co_3_O_4_@MMT group. Statistical analyses were performed by comparing the DSS group with other groups (n = 4), with ∗p < 0.05, ∗∗p < 0.01, ∗∗∗p < 0.001, and ns indicating no significant difference.

To determine whether the therapeutic efficacy of Co_3_O_4_@MMT involves restructuring of the intestinal microbial ecosystem, we profiled the gut microbiota composition in colitic mice following treatment. α-Diversity analysis, assessed through the Shannon and Chao1 indices, revealed that DSS challenge caused a marked reduction in both microbial richness and community evenness—a signature of dysbiosis commonly observed in active inflammatory bowel disease. Notably, Co_3_O_4_@MMT administration effectively restored these diversity metrics to levels approximating those of healthy controls (Fig. 6A and B), suggesting that the nanocomposite re-establishes a favorable ecological niche for microbial colonization. This restoration was further supported by Venn diagram analysis, which demonstrated a substantially larger overlap in operational taxonomic units (OTUs) between the Co_3_O_4_@MMT and Normal groups relative to the DSS group (Fig. S39), indicating that treatment shifts the compositional landscape toward a eubiotic state.

β-Diversity analysis via principal coordinates analysis (PCoA) based on Bray–Curtis distances revealed a distinct segregation of microbial communities between the DSS and Normal cohorts. Strikingly, the Co_3_O_4_@MMT group clustered closely with the Normal group, whereas the DSS group remained isolated (Fig. 6C). This marked convergence toward a healthy community architecture implies that Co_3_O_4_@MMT not only alleviates inflammation but also rectifies the underlying ecological disturbance—a property rarely achieved by conventional anti-inflammatory agents.

At finer taxonomic resolution, Co_3_O_4_@MMT treatment enriched several putatively beneficial taxa, including CAG-239 and CAG-314 (Fig. 6D). At the phylum level, Co_3_O_4_@MMT significantly increased the relative abundances of Bacteroidota and Firmicutes [50], while concurrently suppressing the bloom of Proteobacteria (Fig. 6E–G)—a Gram-negative phylum whose expansion is tightly correlated with mucosal inflammation and endotoxemia in IBD patients [51]. This reciprocal modulation of beneficial and potentially harmful phyla underscores the capacity of Co_3_O_4_@MMT to reconstruct a resilient, anti-inflammatory microbial consortium.

To identify the specific bacterial clades most responsive to the intervention, we performed linear discriminant analysis effect size (LEfSe). This analysis uncovered several taxonomic units that were significantly enriched in the Co_3_O_4_@MMT cohort, notably including CAG-239, CAG-314, and CAG-552 (Fig. 6H and I). These genera have been increasingly recognized in recent metagenomic studies as indicators of a healthy gut ecosystem, and their depletion is frequently linked to colitis progression. The selective enrichment of these organisms by Co_3_O_4_@MMT suggests that the nanocomposite may create an oxidative stress-alleviated, anti-inflammatory luminal microenvironment that favors the outgrowth of obligate anaerobes and saccharolytic bacteria, while suppressing facultative anaerobes adapted to inflamed niches. Collectively, these compositional and ecological shifts demonstrate that Co_3_O_4_@MMT actively remodels the dysbiotic gut microbiota toward a eubiotic configuration, thereby reinforcing the intestinal mucosal barrier and contributing to the sustained amelioration of experimental colitis.

## 4 Conclusion

This study successfully developed an orally deliverable nanozyme platform, Co_3_O_4_@MMT, by anchoring catalytically active cobalt oxide nanoparticles onto exfoliated montmorillonite nanosheets. The platform is engineered to overcome key challenges in oral nanotherapy, exhibiting efficient multi-enzyme-mimetic ROS-scavenging activity, enhanced stability throughout the gastrointestinal tract, and electrostatic targeting to inflamed colonic mucosa. These integrated properties enable Co_3_O_4_@MMT to execute a coordinated therapeutic strategy against colitis, simultaneously performing antioxidant, anti-inflammatory, barrier-repairing, and microbiota-modulating functions. Comprehensive in vitro and in vivo evaluations confirmed that oral administration of Co_3_O_4_@MMT effectively alleviates oxidative stress, suppresses the production of key pro-inflammatory cytokines, and promotes the restoration of epithelial tight junctions, leading to significant mitigation of disease severity. Furthermore, 16S rRNA sequencing analysis demonstrated that treatment reshapes the dysbiotic gut microbiota towards a healthier compositional state. Mechanistic investigations revealed that the material modulates immune responses and reactivates cellular repair programs, thereby disrupting the vicious cycle between oxidative damage and chronic inflammation. Collectively, Co_3_O_4_@MMT represents a multifunctional oral nanotherapeutic that concurrently addresses the interconnected pathological pillars of IBD—oxidative stress, inflammation, barrier disruption, and microbial imbalance. This work provides a comprehensive and translatable approach to achieving sustained mucosal healing and immune homeostasis, offering a promising new direction for the clinical management of inflammatory bowel diseases.

## CRediT authorship contribution statement

**Yinxi Li:** Data curation, Formal analysis, Investigation, Methodology, Software, Validation, Writing – original draft. **Qinxuan Zhou:** Data curation, Formal analysis, Methodology. **Yuxuan She:** Methodology, Validation. **Bin Lu:** Conceptualization, Data curation, Formal analysis. **Mengting Wu:** Data curation, Formal analysis. **Tianhao Chen:** Methodology, Software. **Pengcheng Ye:** Software, Validation. **Li Yu:** Formal analysis, Methodology, Software. **Wenzhao Liu:** Funding acquisition, Software, Validation. **Hui Deng:** Conceptualization, Funding acquisition, Writing – review & editing. **Xiaowen Hu:** Conceptualization, Funding acquisition, Validation, Writing – review & editing.

## Declaration of competing interest

The authors declare that they have no known competing financial interests or personal relationships that could have appeared to influence the work reported in this paper.

## Data Availability

Data will be made available on request.
