# Supplementary material for: An orally deliverable Co3O4@MMT nanozyme platform for inflammatory bowel disease via ROS scavenging, barrier repair, and mucosal homeostasis regulation
Source: Mater Today Bio. 2026 Jun 1;38:103310. doi: 10.1016/j.mtbio.2026.103310 (PMC13253145; doi:10.1016/j.mtbio.2026.103310)
Supplement: Multimedia component 1 [file mmc1.docx]

**Supporting Information**

**An Orally Deliverable Co_3_O_4_@MMT Nanozyme Platform for Inflammatory bowel disease via ROS Scavenging, Barrier Repair, and Mucosal Homeostasis Regulation**

Supplementary Figures and Tables


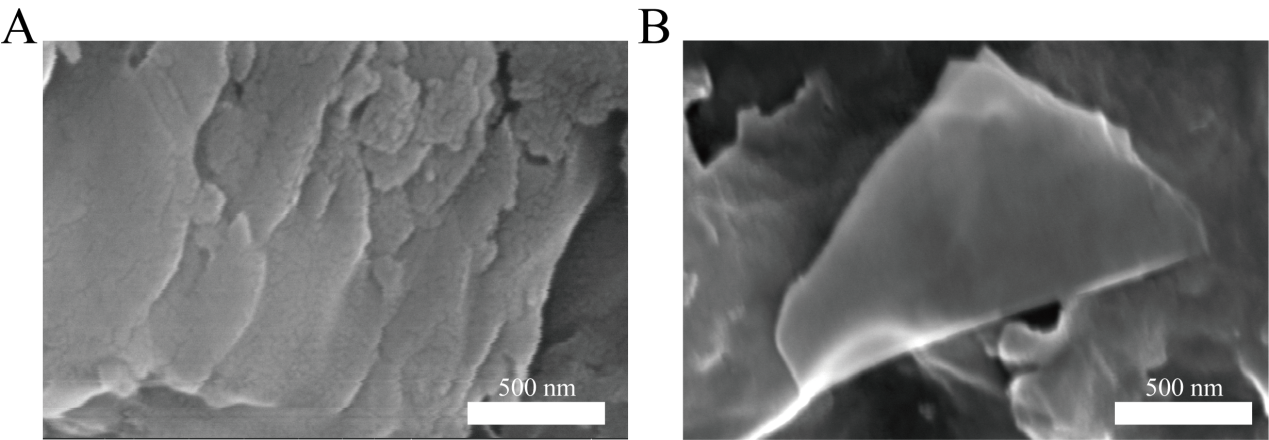


Fig. S1 SEM images of montmorillonite (MMT): (A) pristine MMT and (B) ammonia‑exfoliated MMT nanosheets.


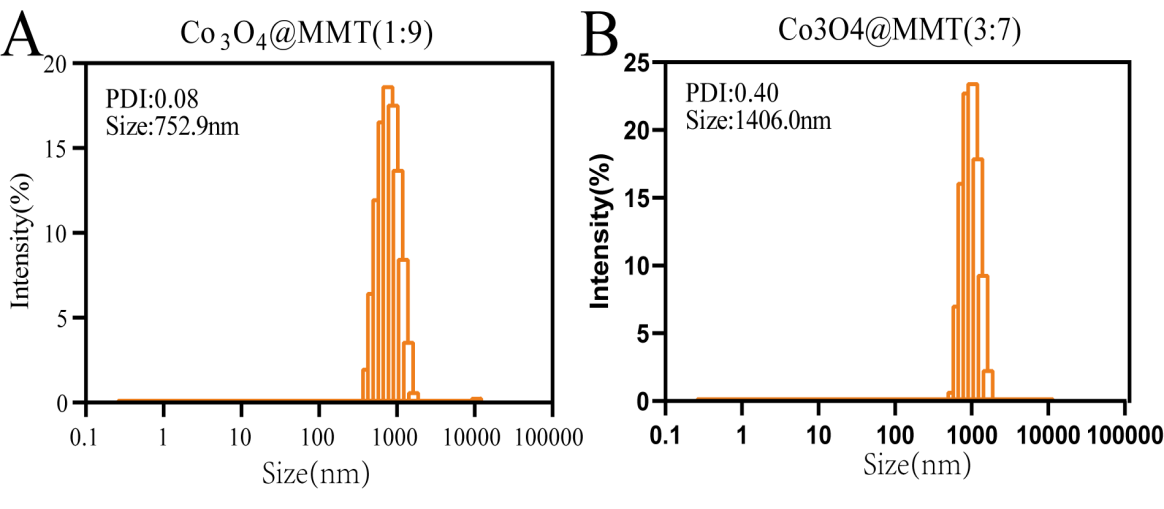


Fig. S2 Hydrodynamic size and polydispersity index (PDI) of (A) Co_3_O_4_@MMT (1:9) and (B) Co_3_O_4_@MMT (3:7).

Table S1 Comparative hydrodynamic size and PDI of Co_3_O_4_@MMT nanocomposites with different mass ratios.

|  | Co_3_O_4_@MMT(1:9) | Co_3_O_4_@MMT(2:8) | Co_3_O_4_@MMT(3:7) |
| --- | --- | --- | --- |
| Size(nm) | 752.9 | 859.6 | 1406 |
| PDI | 0.08 | 0.06 | 0.4 |


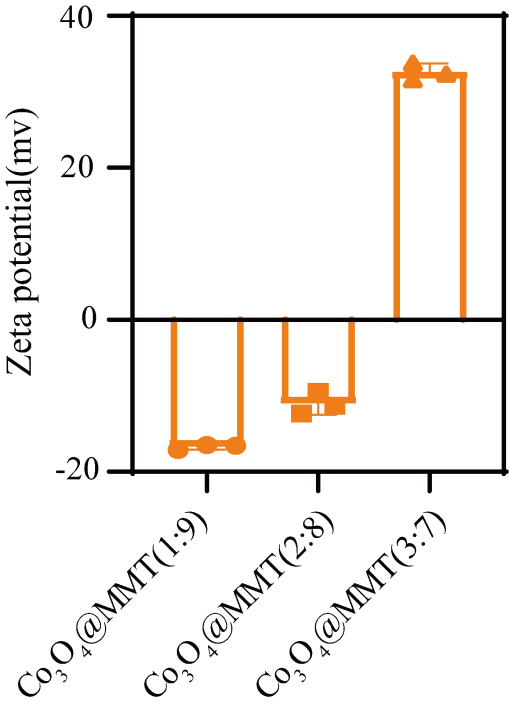


**Fig. S3** Zeta potential of Co_3_O_4_@MMT formulations with different mass ratios.


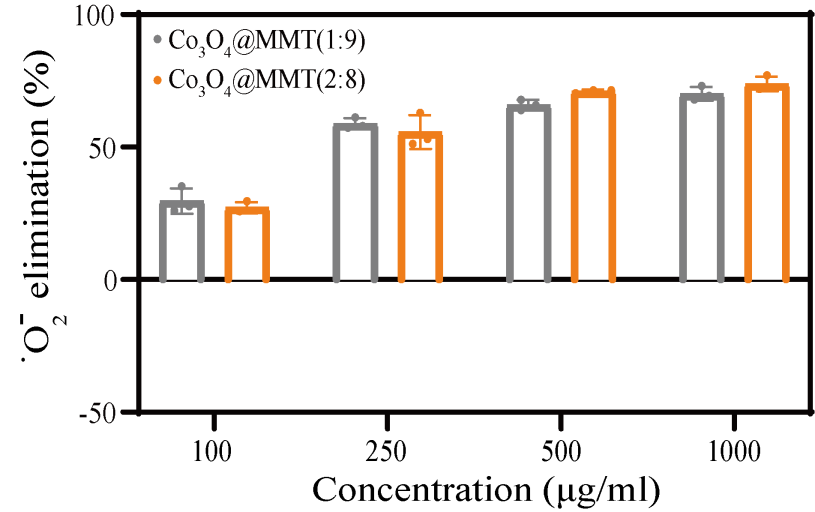


Fig. S4 SOD-mimetic activities of Co_3_O_4_@MMT (1:9) and Co_3_O_4_@MMT (2:8).


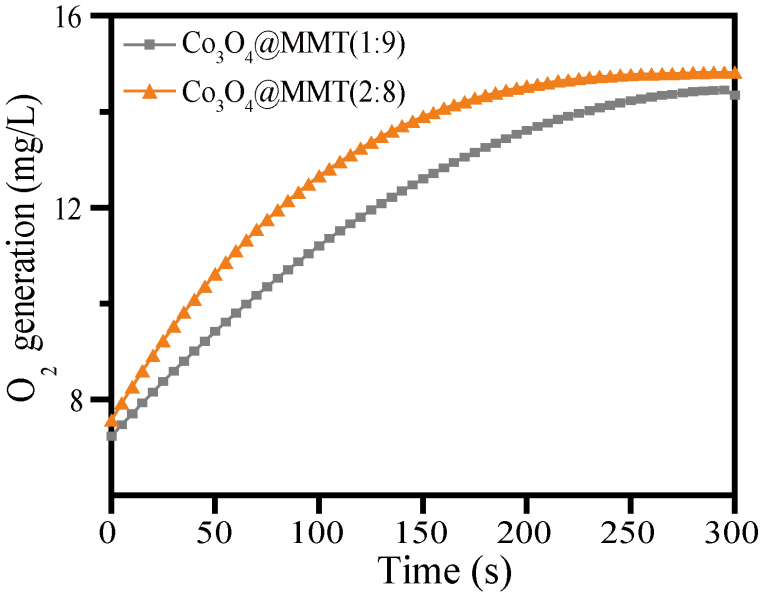


Fig. S5 CAT-mimetic activities of Co_3_O_4_@MMT (1:9) and Co_3_O_4_@MMT (2:8).


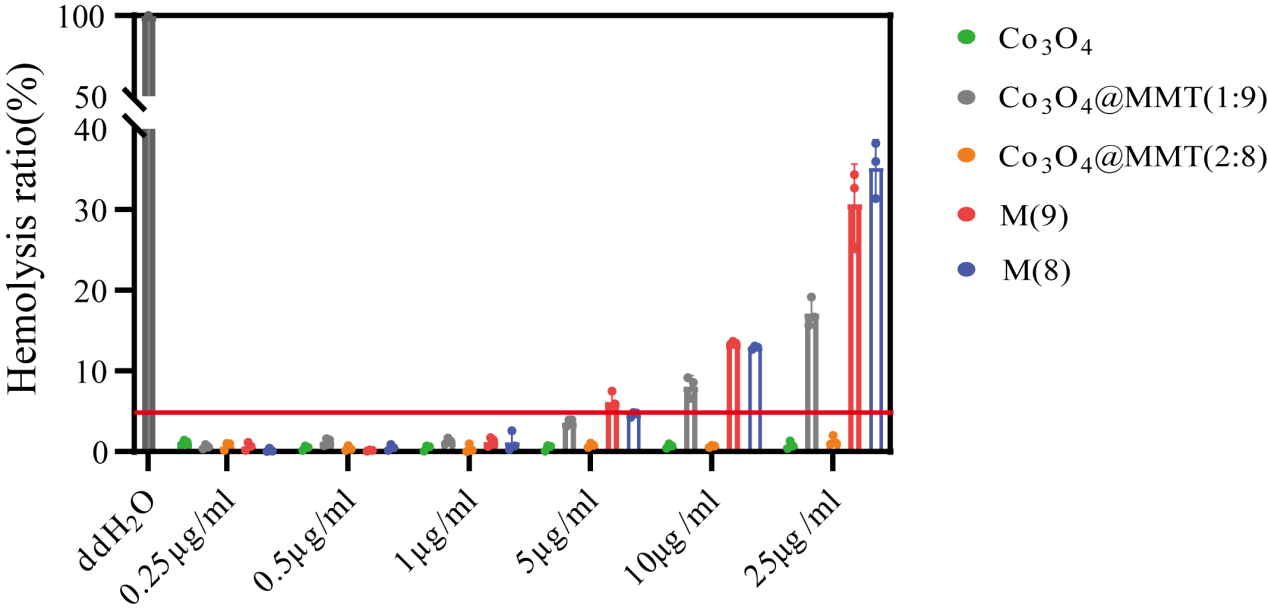


Fig. S6 Hemolysis rates of Co_3_O_4_@MMT nanocomposites at different mass ratios.


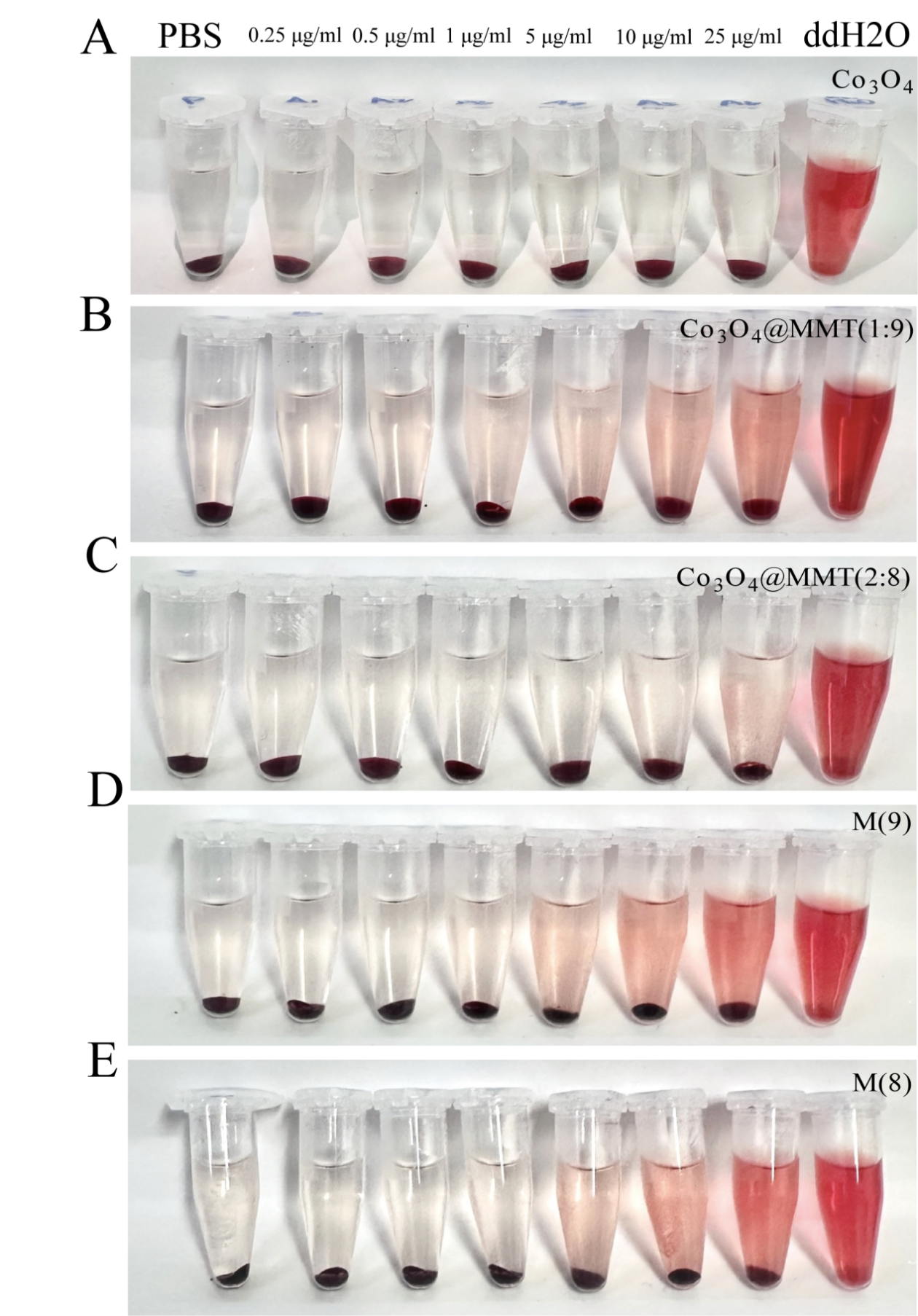


Fig. S7 Representative images of hemocompatibility assays for Co_3_O_4_@MMT with different mass ratios.


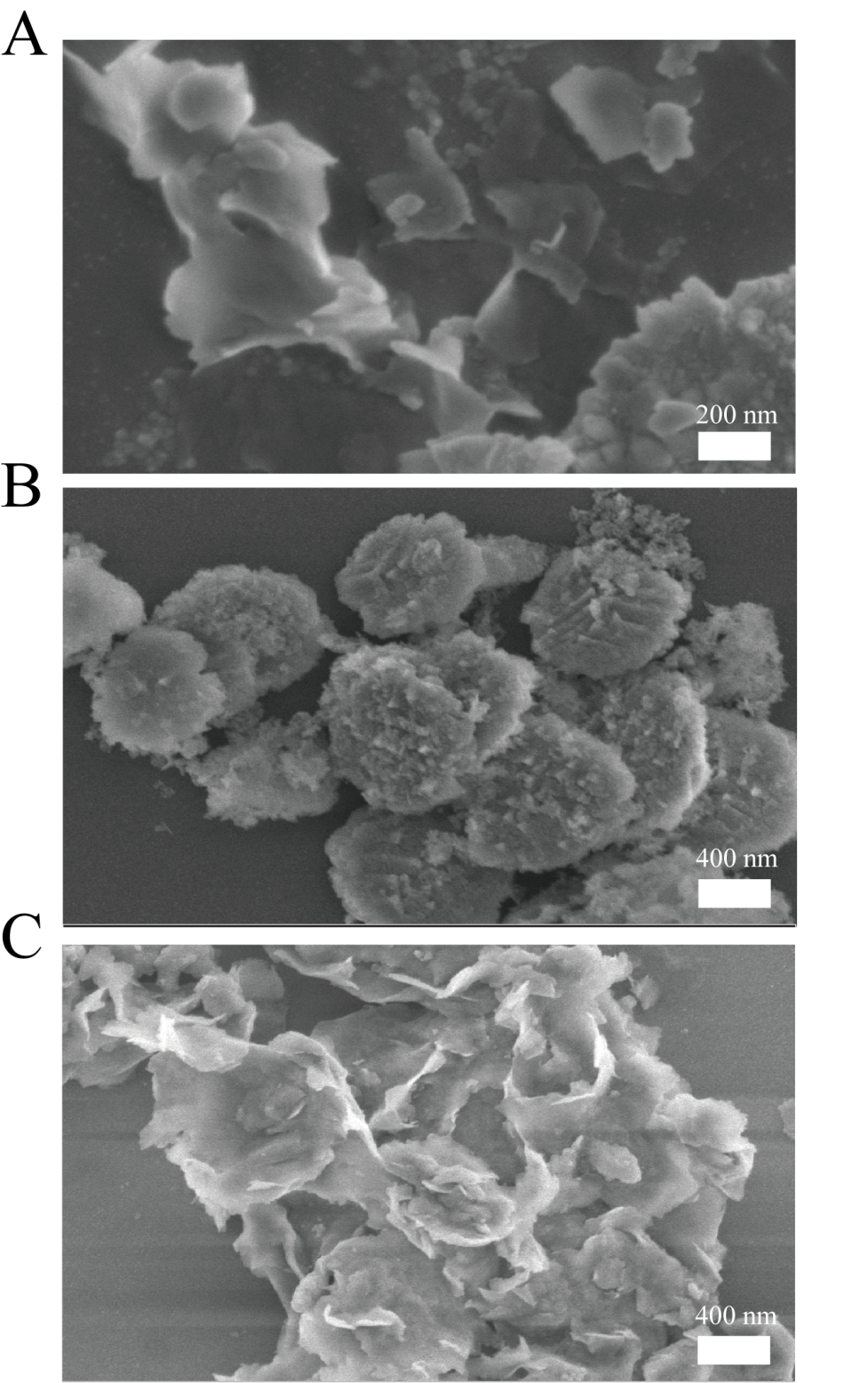


Fig. S8 SEM images of MMT, Co_3_O_4_, and Co_3_O_4_@MMT.


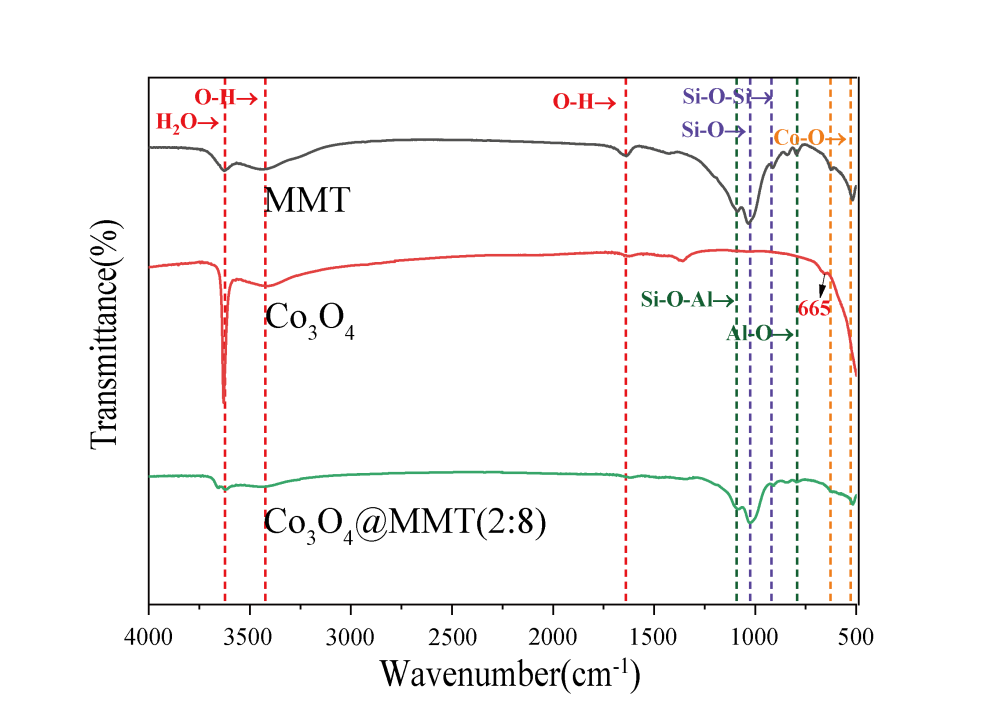


Fig. S9 Fourier‑transform infrared (FTIR) spectra of MMT, Co_3_O_4_, and Co_3_O_4_@MMT.


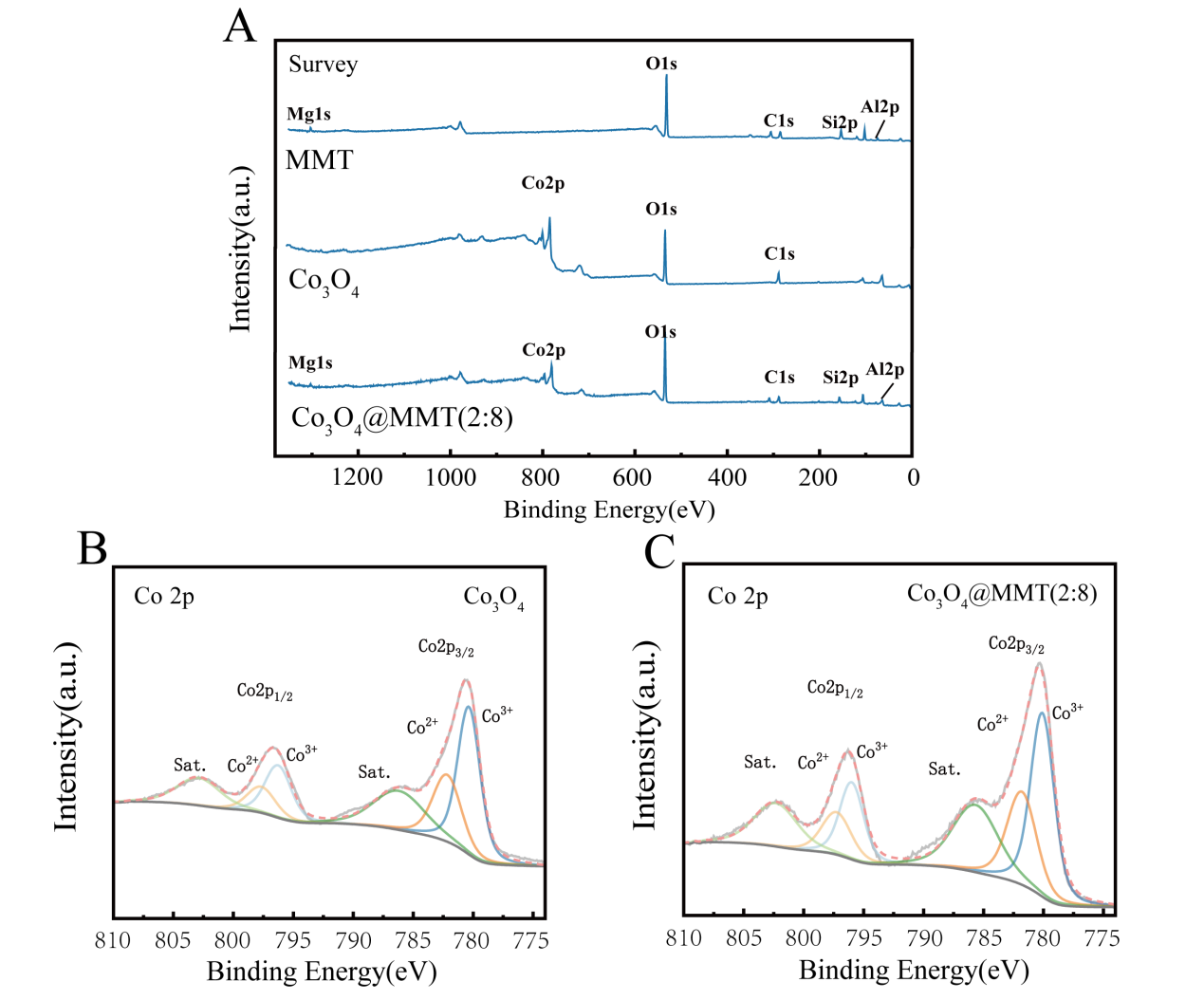


Fig. S10 X‑ray photoelectron spectroscopy (XPS) spectra of MMT, Co_3_O_4_, and Co_3_O_4_@MMT.


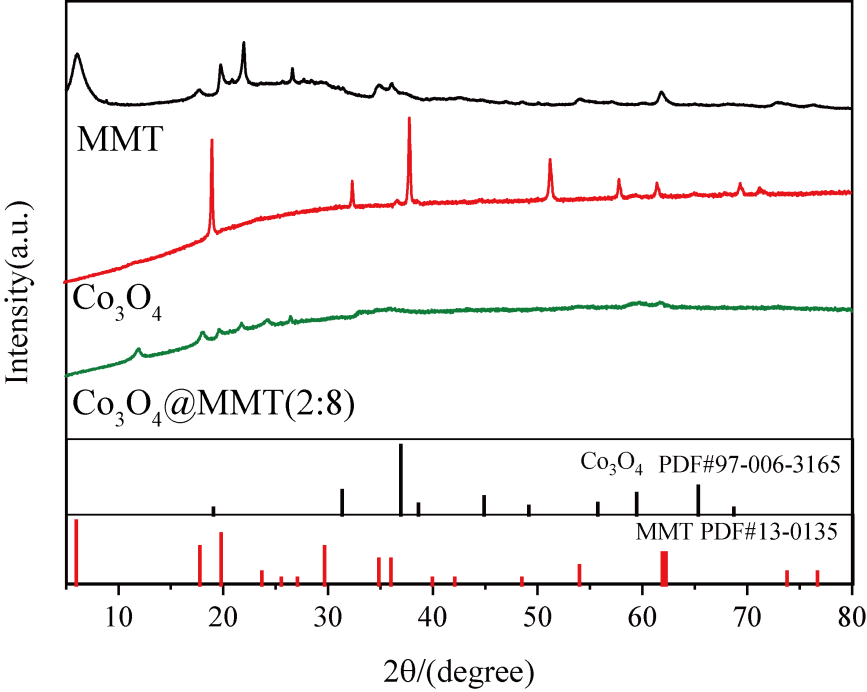


Fig. S11 XRD patterns of MMT, Co_3_O_4_, and Co_3_O_4_@MMT.


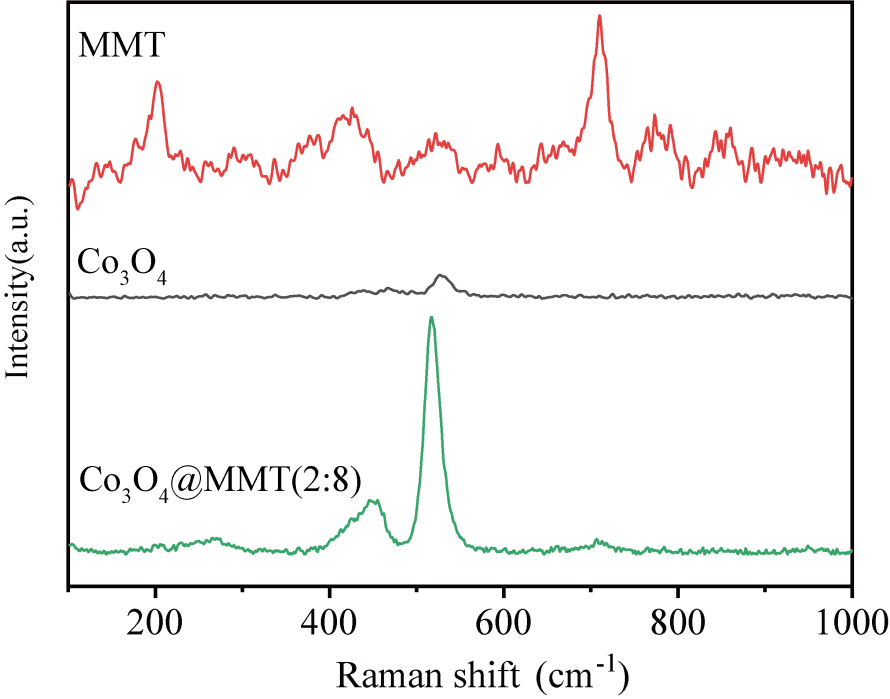


Fig. S12 Raman spectra of MMT, Co_3_O_4_, and Co_3_O_4_@MMT.


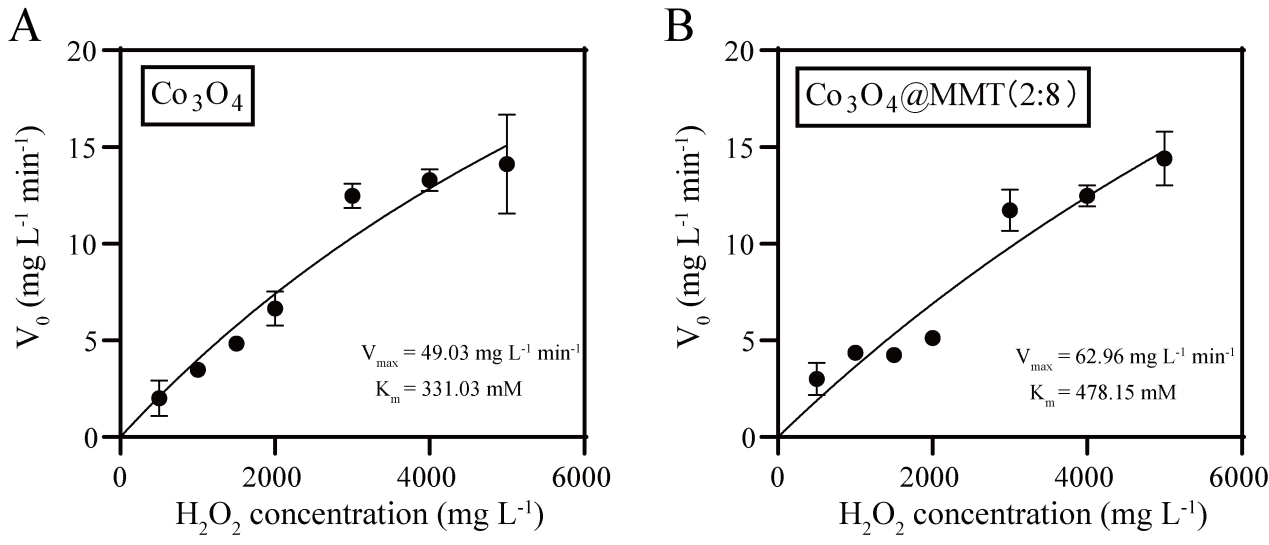


Fig. S13 Steady-state kinetic analysis of nanozyme activity: Typical Michaelis-Menten curves for determining the kinetic constants of Co_3_O_4_ and Co_3_O_4_@MMT with H_2_O_2_ as the substrate.


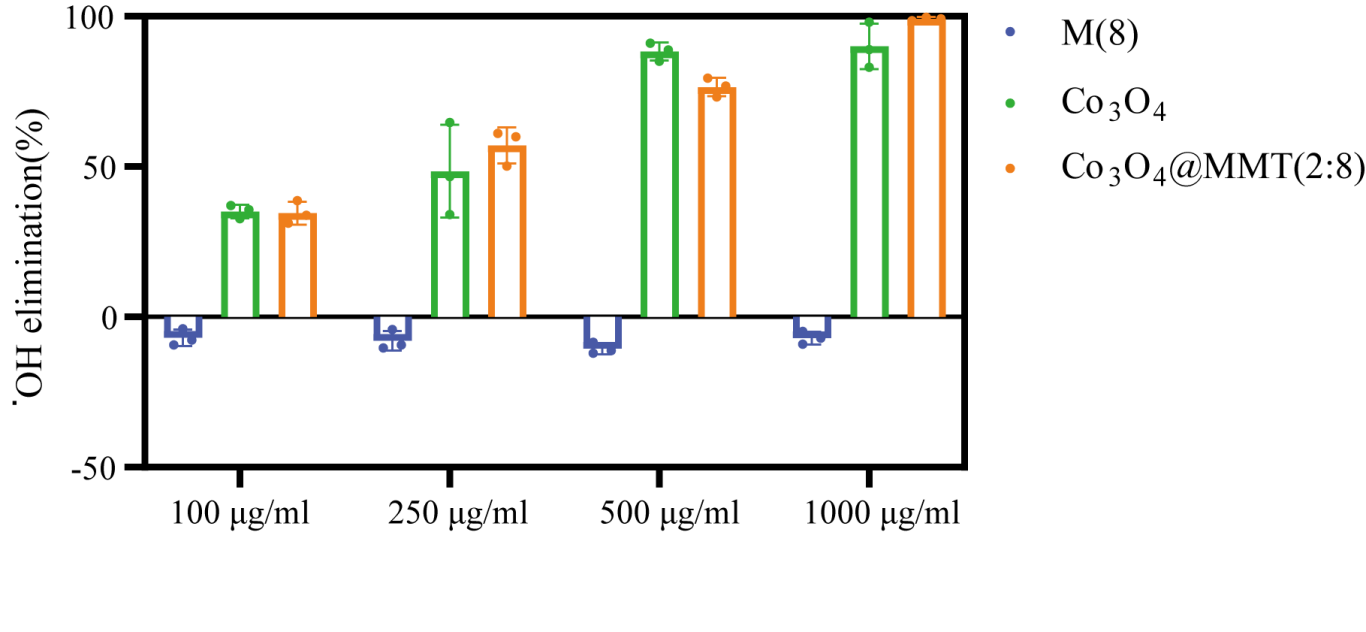


Fig. S14 Hydroxyl radical scavenging activity of MMT, Co₃O₄, and Co₃O₄@MMT.


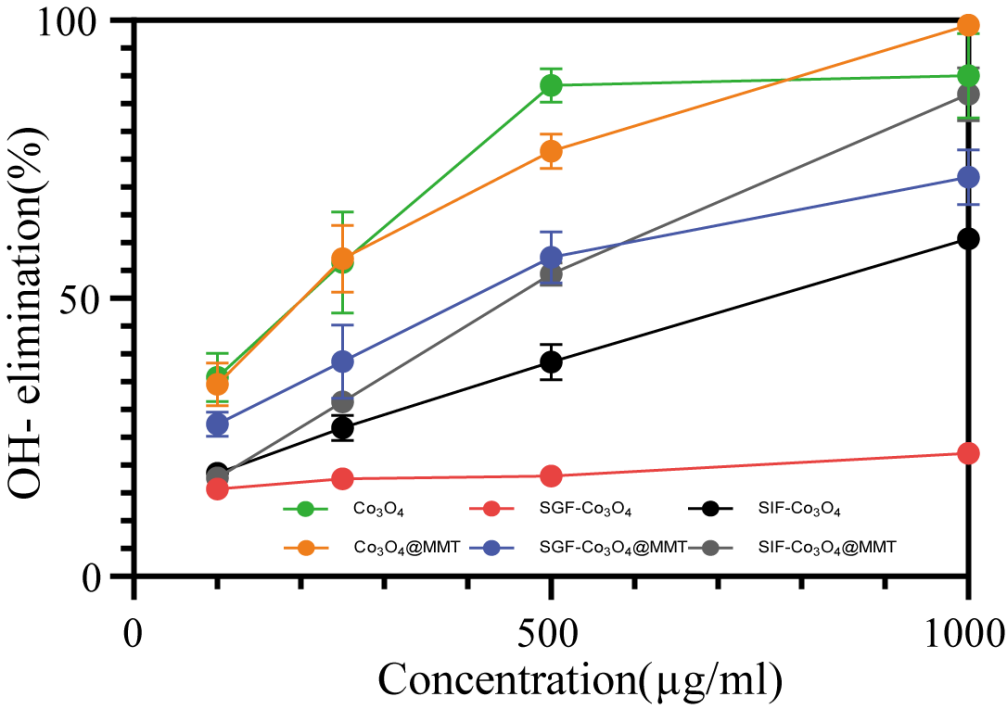


Fig. S15 •OH scavenging activity of MMT, Co₃O₄, and Co₃O₄@MMT after treatment with simulated gastric fluid (SGF) or simulated intestinal fluid (SIF).


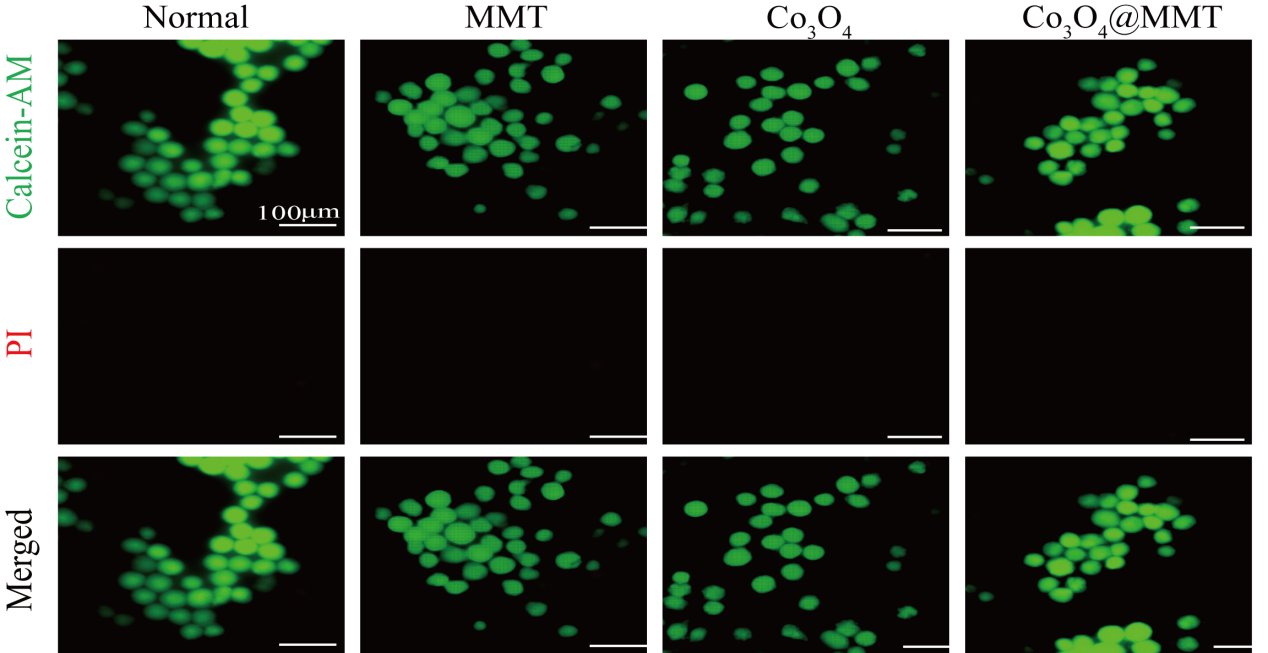


Fig. S16 Live/dead staining of RAW 264.7 macrophages after treatment.


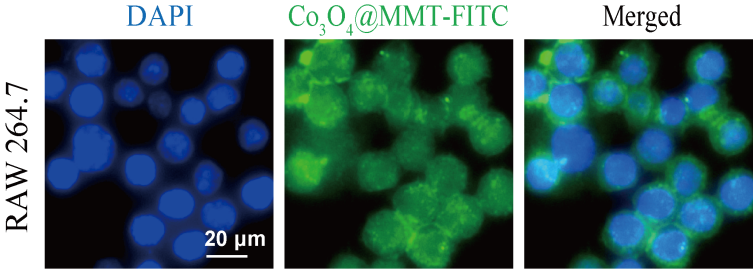


Fig. S17 Cellular uptake of FITC-labeled Co_3_O_4_@MMT in RAW 264.7 cells.


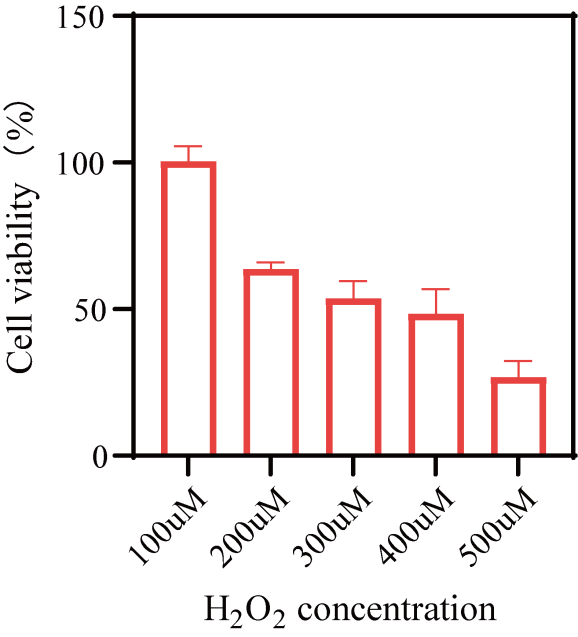


Fig. S18 Determination of the optimal H_2_O_2_ concentration for establishing an oxidative stress model in RAW 264.7 cells using CCK-8 assay.


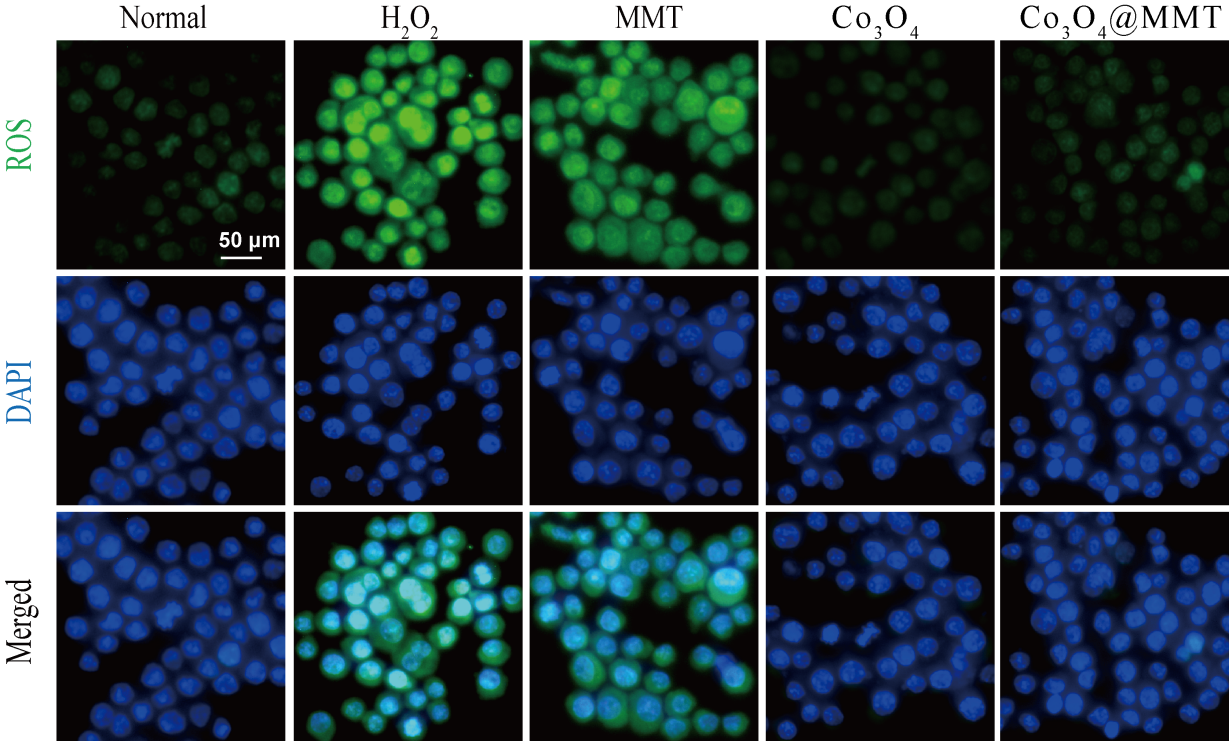


Fig. S19 Intracellular ROS levels in H_2_O_2_-stimulated RAW 264.7 cells, as detected by DCFH-DA staining.


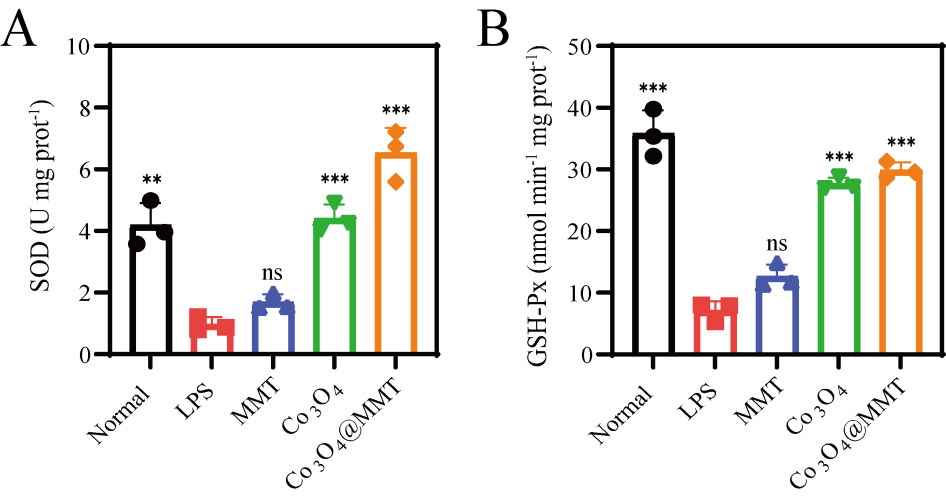


Fig. S20 Determination of endogenous antioxidant enzyme activities (e.g., SOD and GSH-Px) in RAW 264.7 cells.


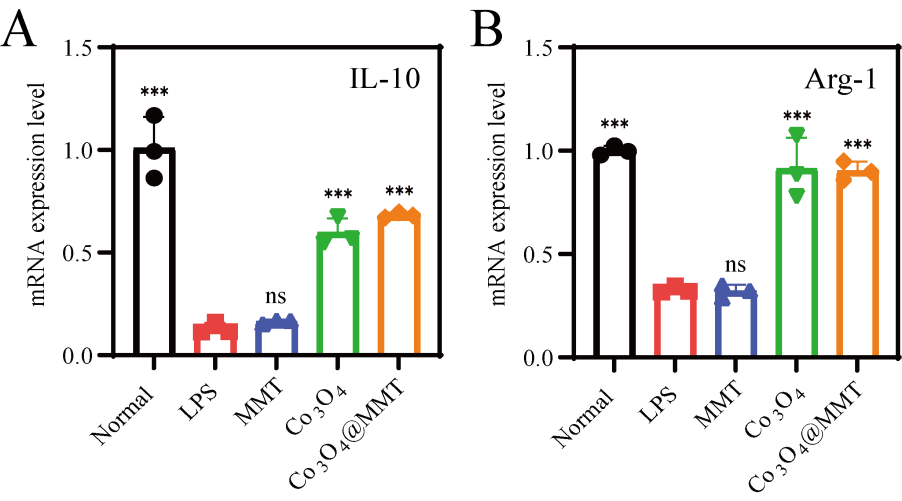


Fig. S21 Relative mRNA expression levels of anti-inflammatory cytokines (IL-10 and Arg-1) in RAW 264.7 cells, as determined by RT-qPCR.


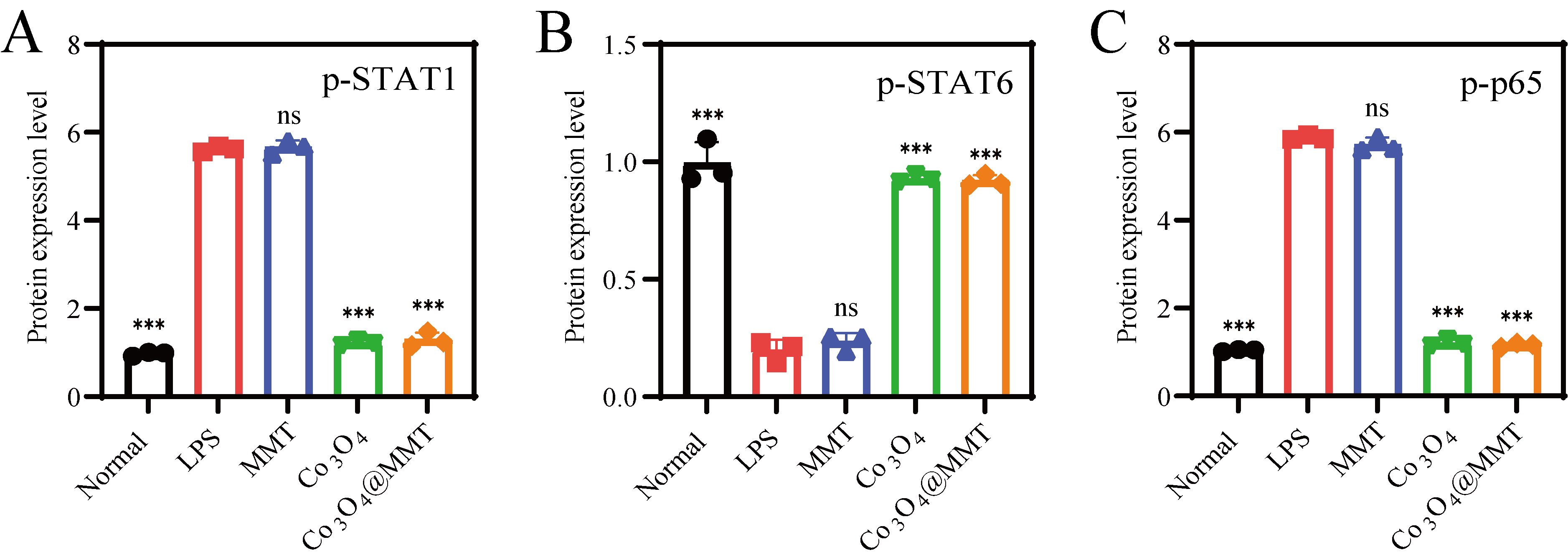


Fig. S22 Protein expression levels of p-STAT1, p-STAT6, and p-p65 in RAW 264.7 cells.


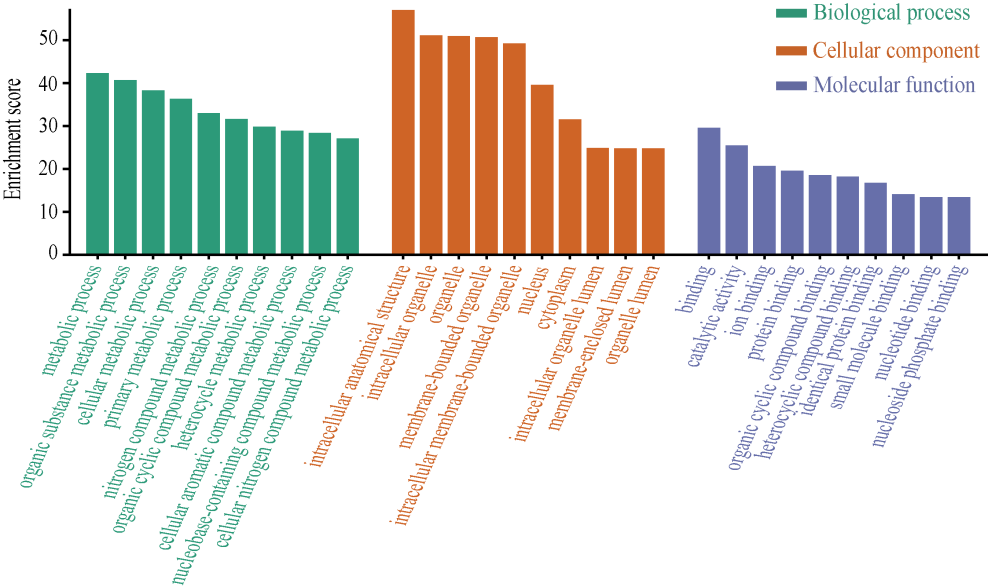


Fig. S23 GO enrichment analysis of DEGs in RAW 264.7 cells.


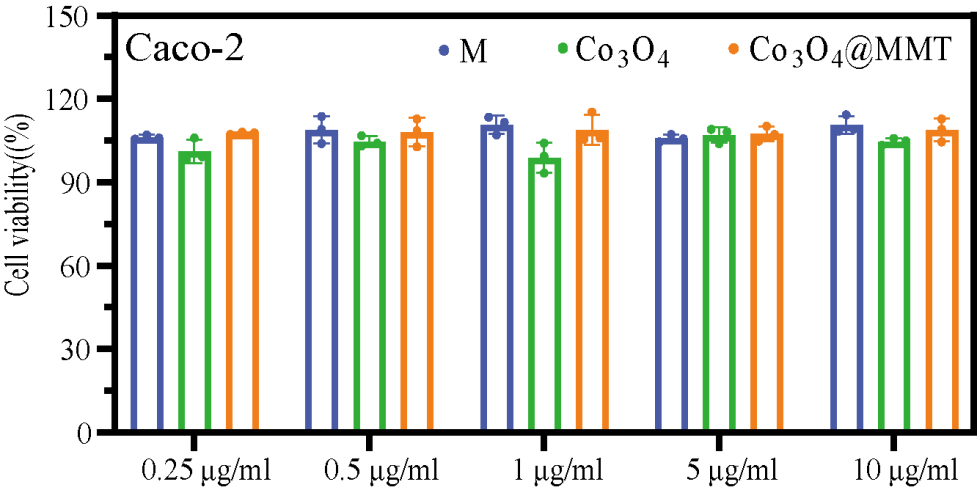


Fig. S24 Biocompatibility of the materials toward Caco‑2 cells assessed by CCK‑8 assay.


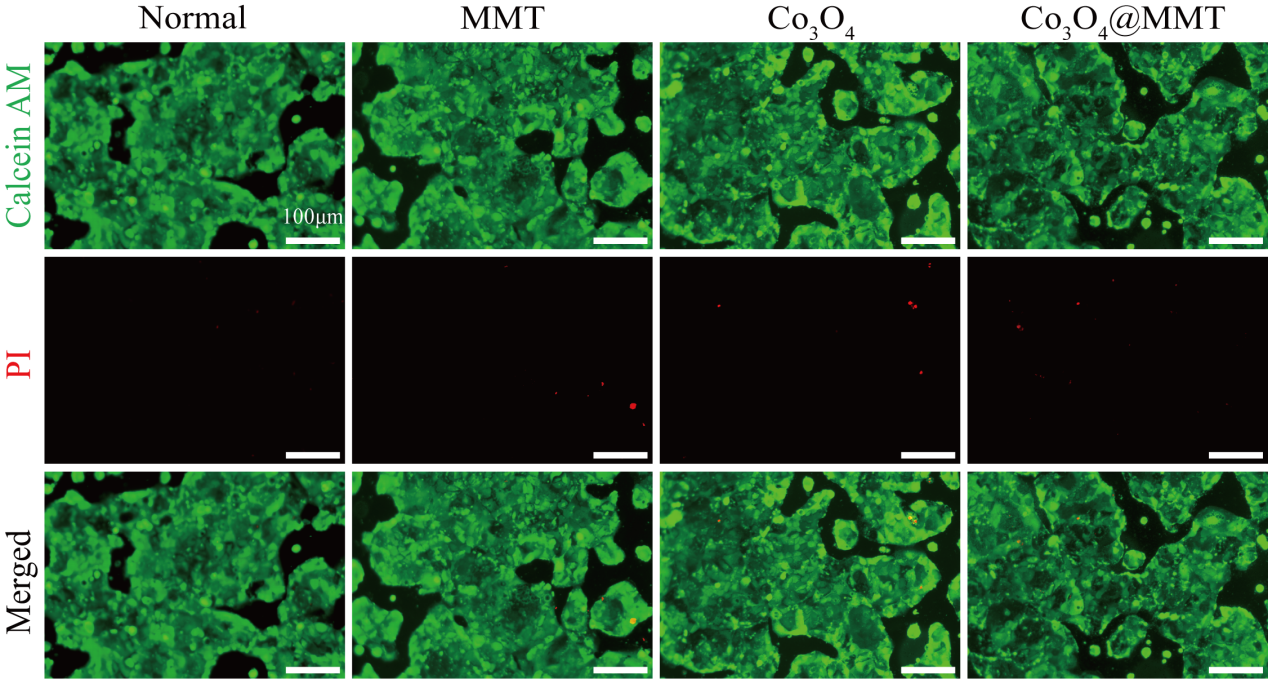


Fig. S25 Live/dead staining of Caco‑2 cells after treatment.


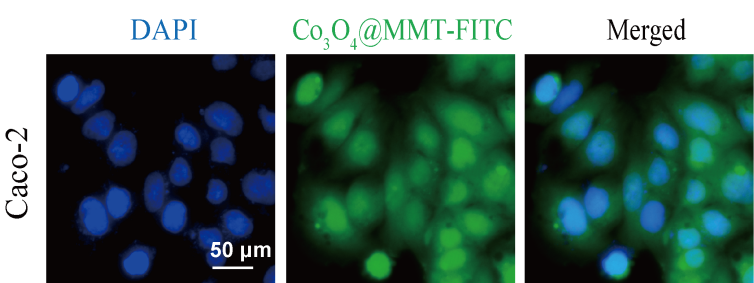


Fig. S26 Cellular uptake of FITC-labeled Co_3_O_4_@MMT in Caco-2 cells.


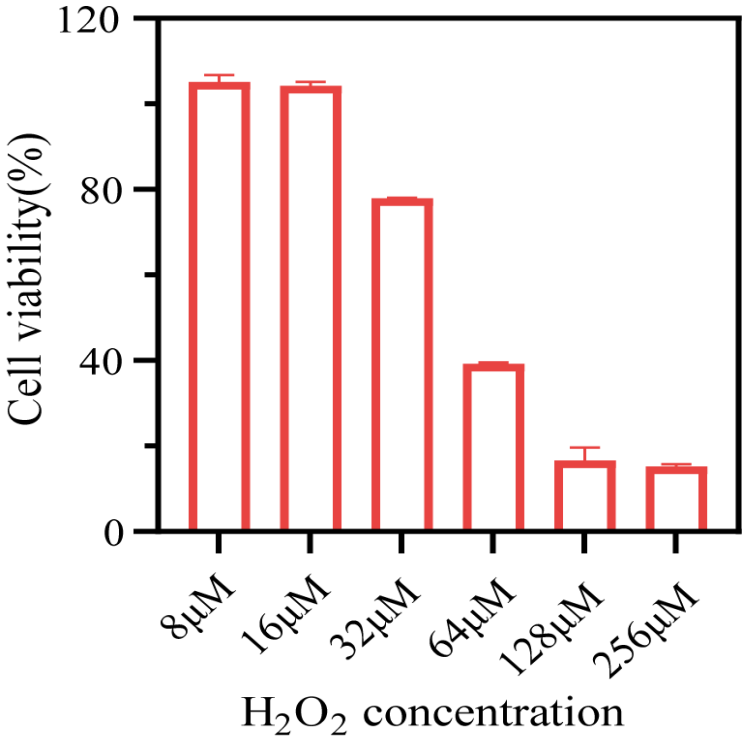


Fig. S27 Determination of the optimal H_2_O_2_ concentration for establishing an oxidative stress model in Caco‑2 cells using CCK‑8 assay.


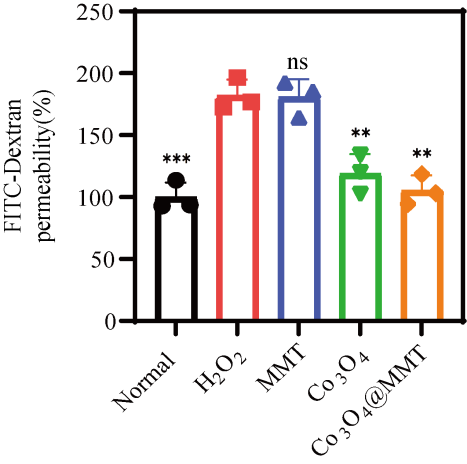


Fig. S28 Evaluation of intestinal barrier integrity via FITC-dextran permeability assay.


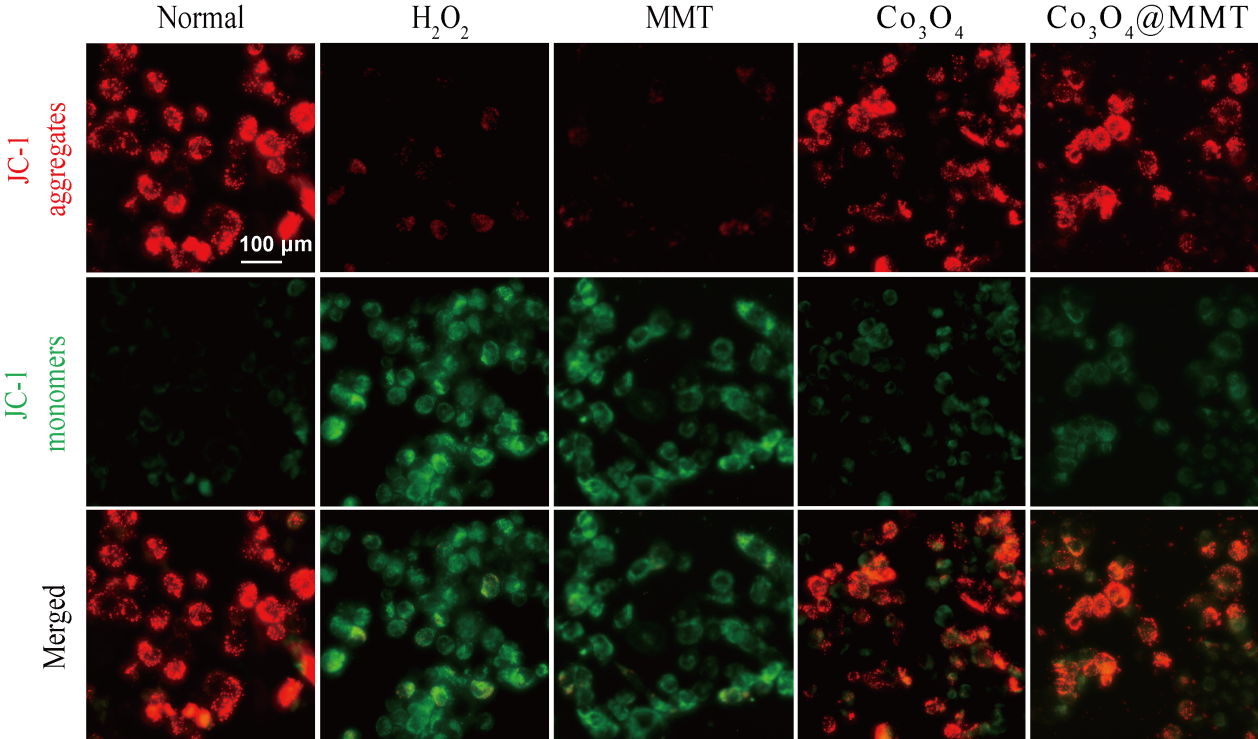


Fig. S29 Protective effect of Co_3_O_4_@MMT on the mitochondrial membrane potential in Caco-2 cells.


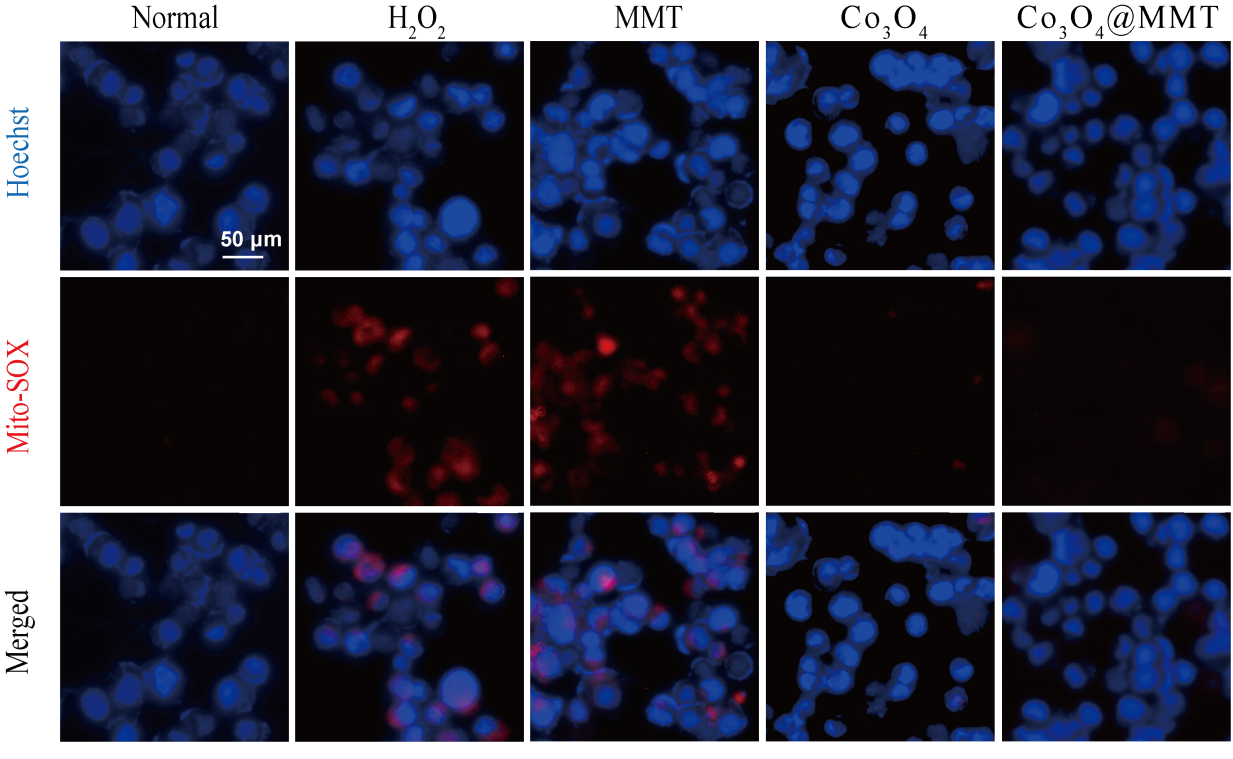


Fig. S30 Mitochondrial ROS scavenging capacity of Co_3_O_4_@MMT in Caco-2 cells.


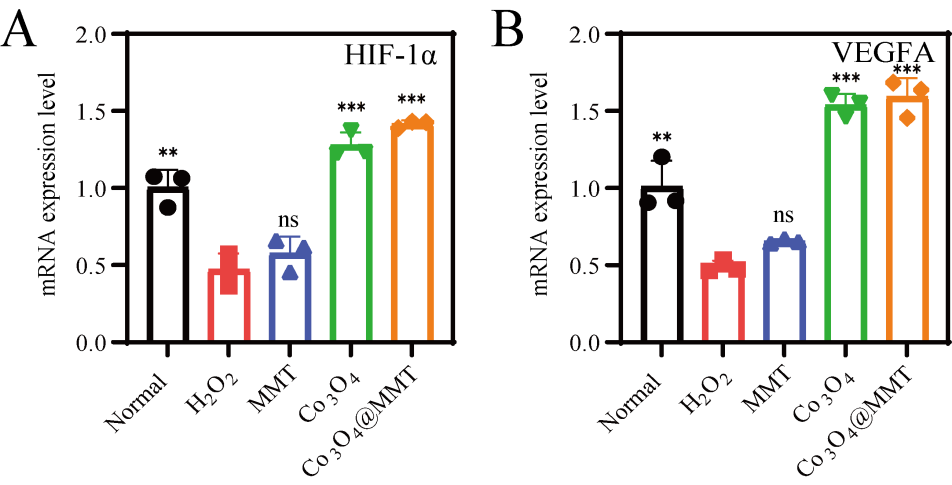


Fig. S31 Relative mRNA expression levels of HIF-1α and VEGFA in Caco-2 cells, as determined by RT-qPCR.


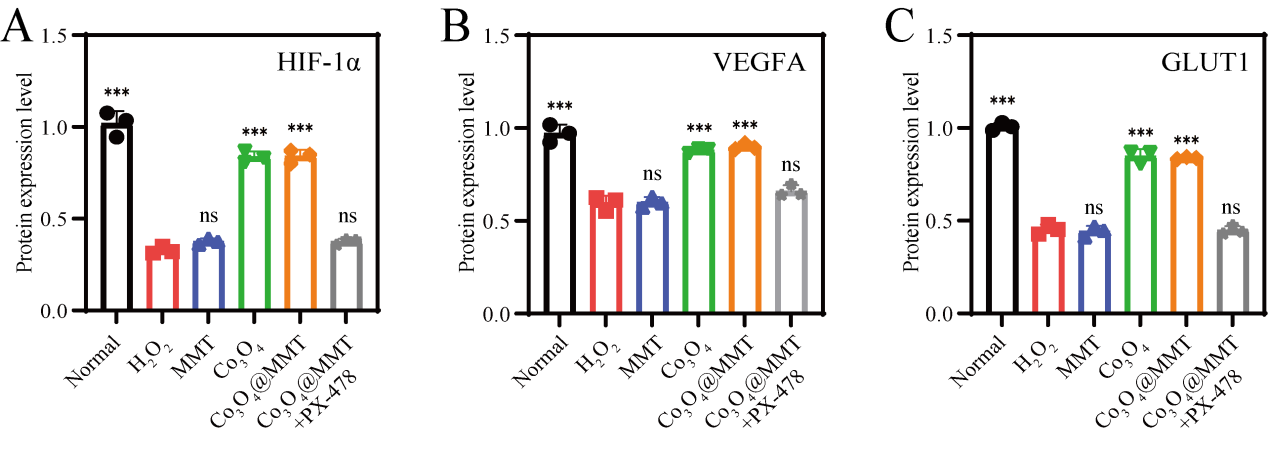


Fig. S32 Protein expression levels of HIF-1α, VEGFA and GLUT1 in Caco-2 cells.


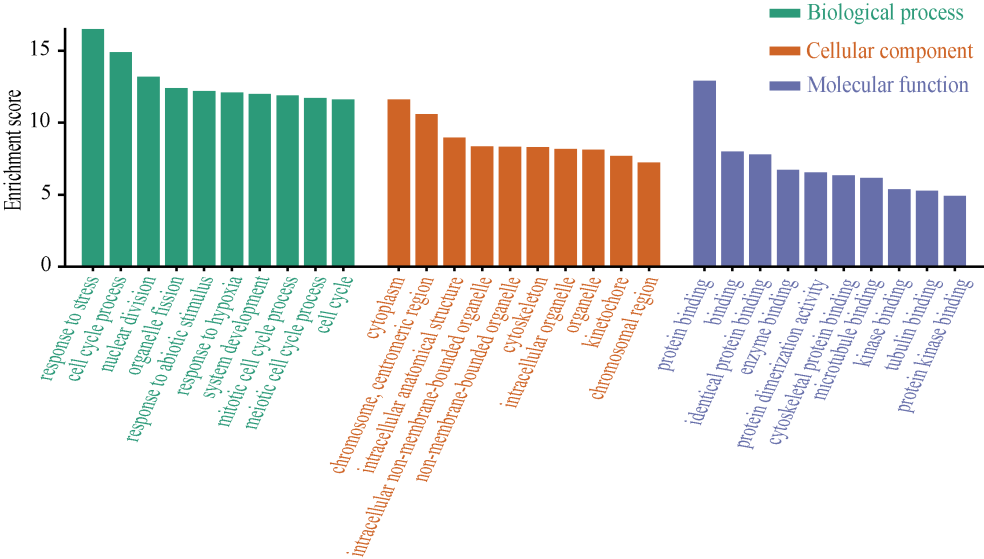


Fig. S33 GO enrichment analysis of DEGs in Caco-2 cells.


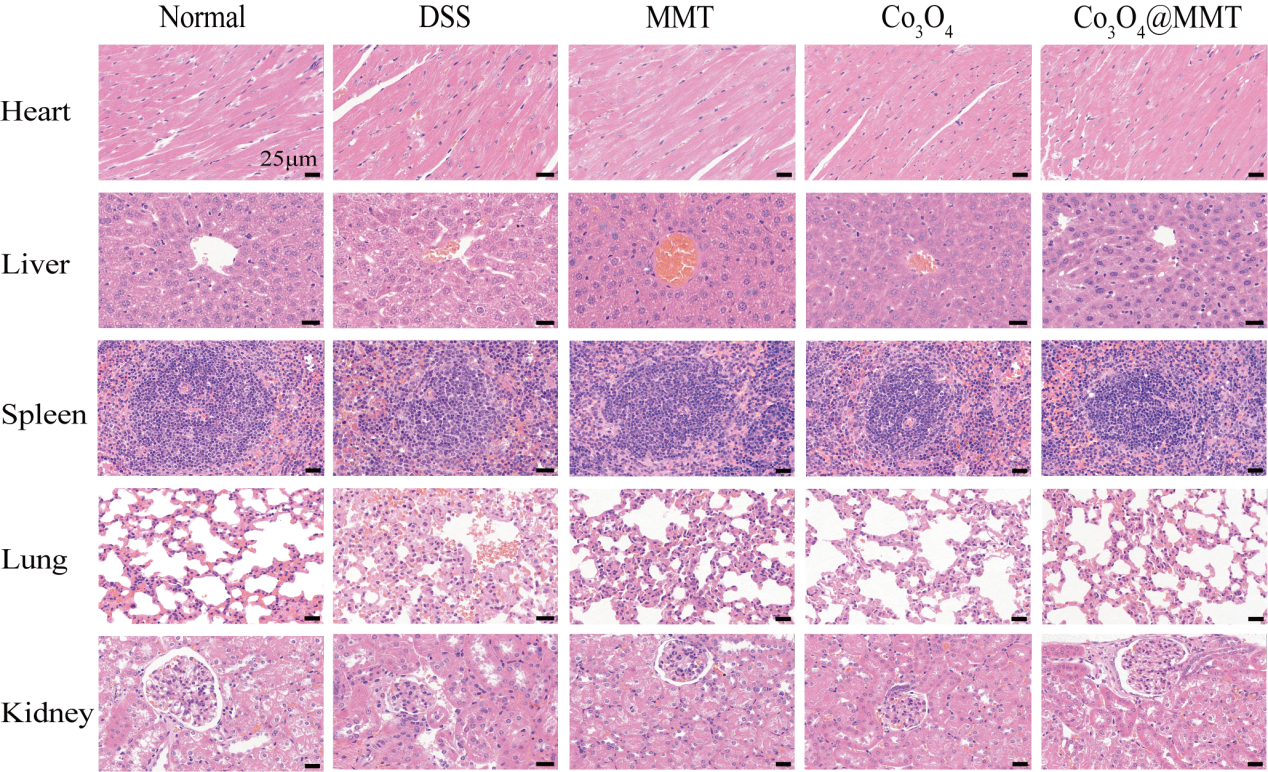


Fig. S34 H&E staining of major organs (heart, liver, spleen, lung, kidney) from mice.


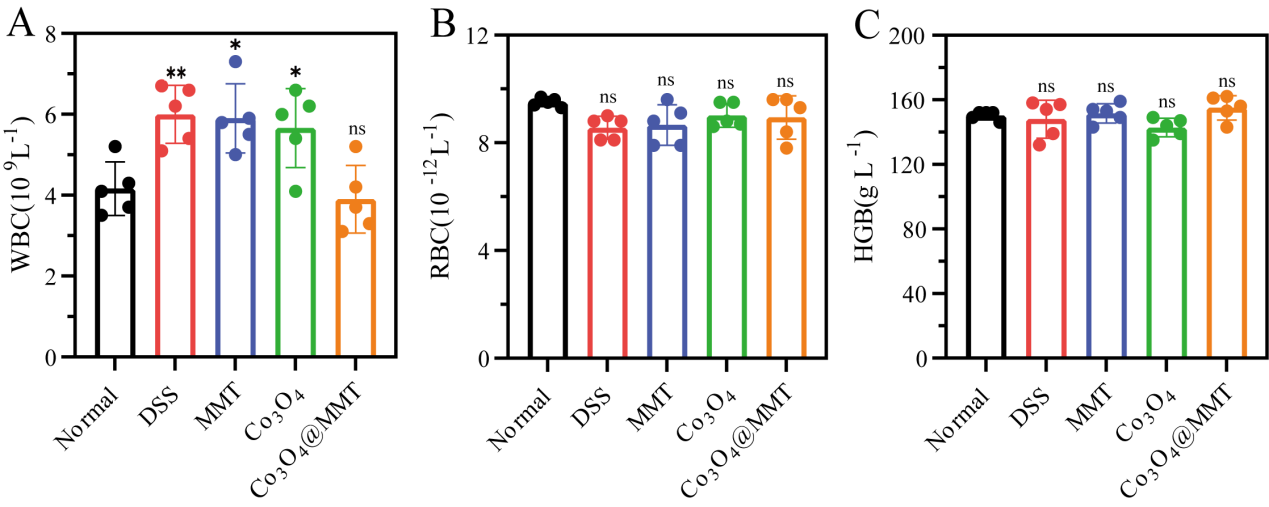


Fig. S35 Hematological parameters of treated mice.


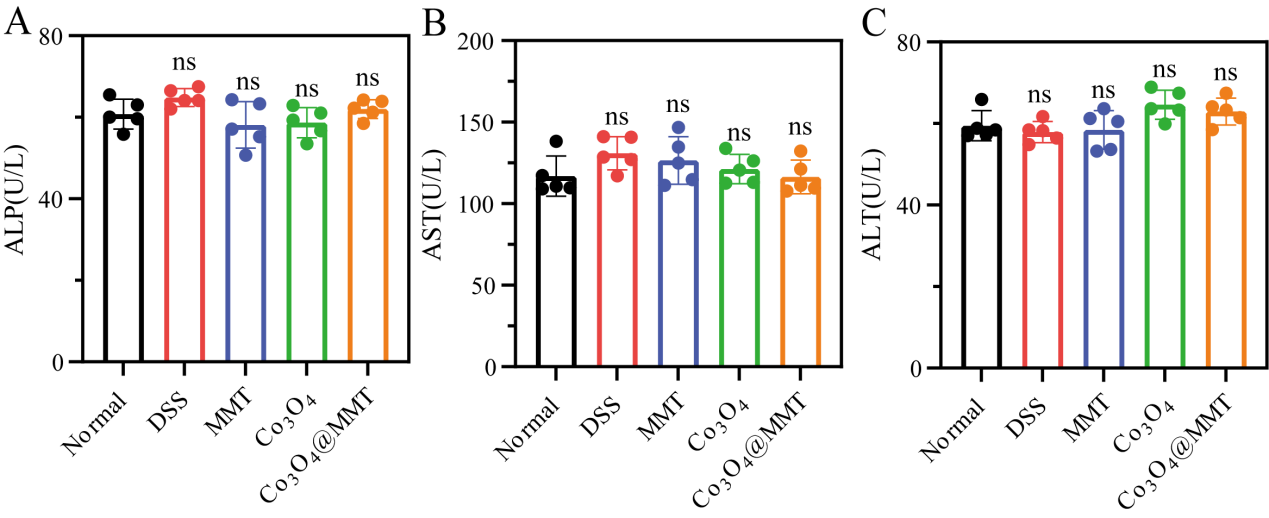


Fig. S36 Serum biochemical indicators of liver function.


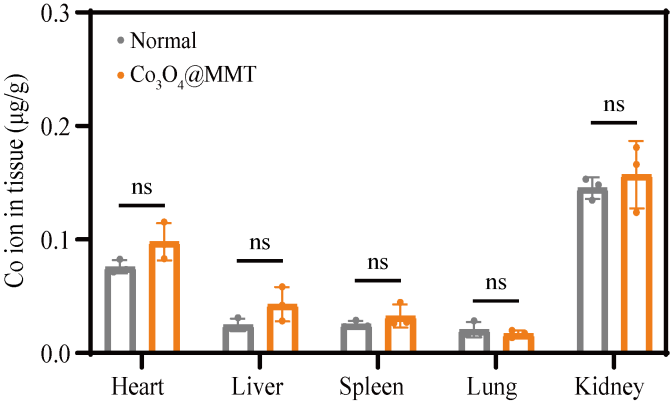


Fig. S37 Co ion distribution in major organs of Co_3_O_4_@MMT-treated UC mice and normal mice after 28 days of treatment. Statistical analyses were performed by comparing the Co_3_O_4_@MMT group with the normal group (n = 3); with *p < 0.05, **p < 0.01, ***p < 0.001, and ns indicating no significant difference.


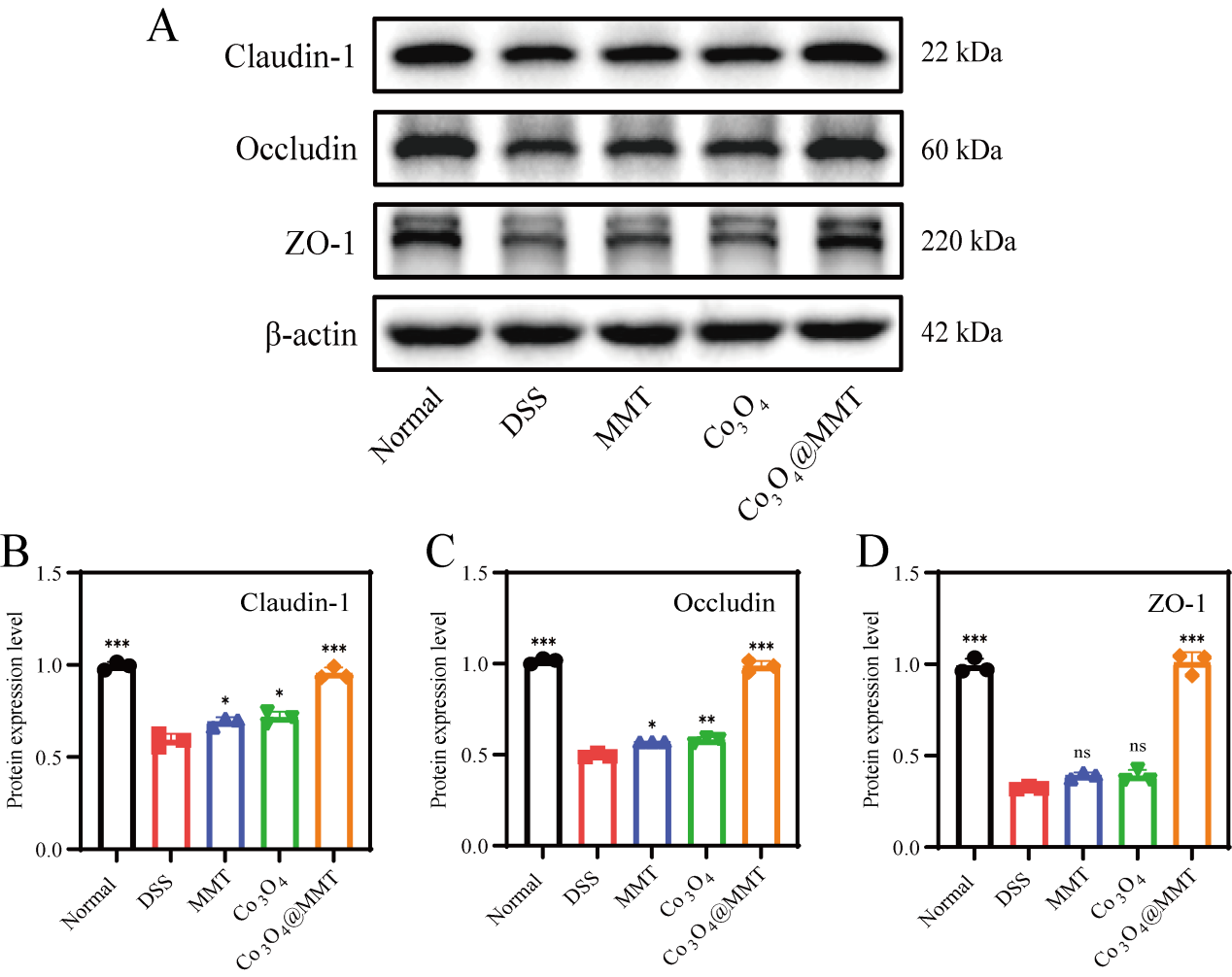


Fig. S38 Protein expression levels of tight junction proteins (Claudin-1, Occludin, and ZO-1) in colonic tissue


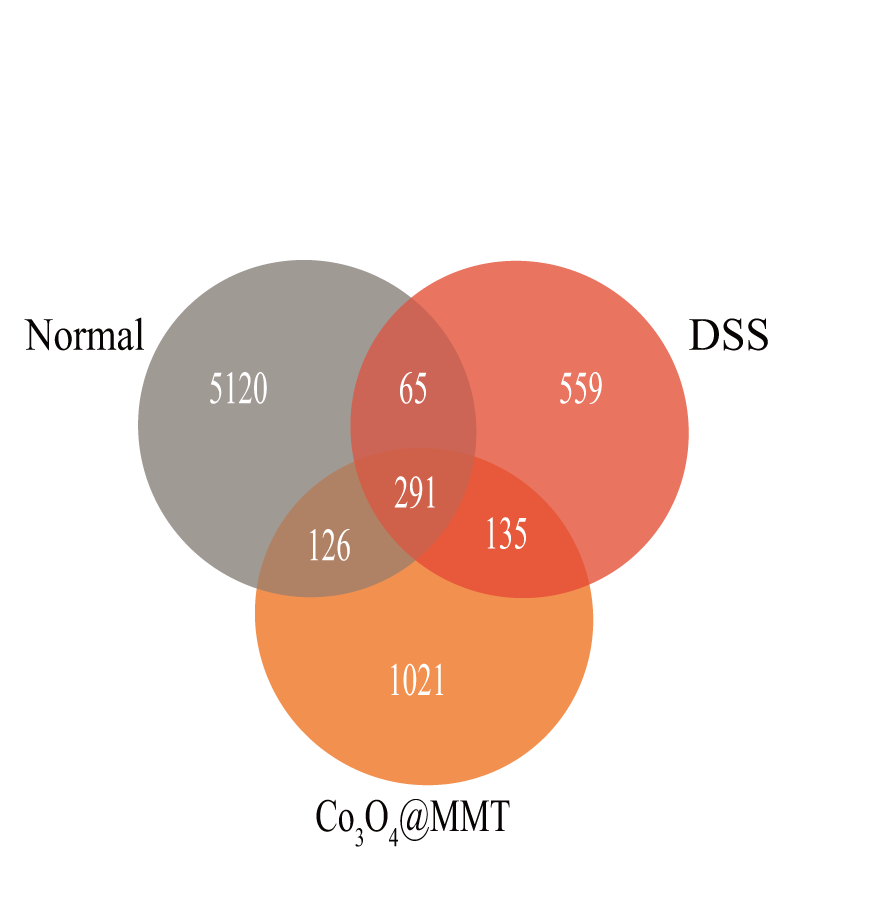


Fig. S39 Venn diagram analysis of gut microbiota from different experimental groups.

Table S2. The Primer sequence of genes used in the study

| Gene | Forward primer sequence (5’-3’) | Reverse primer sequence (3’-5’) |
| --- | --- | --- |
| HO-1 | AAGACTGCGTTCCTGCTCAAC | AAAGCCCTACAGCAACTGTCG |
| TNF-α | TCCAGGCGGTGCCTATGTC | CCTCCACTTGGTGGTTTGTGA |
| IL-1β | TGGAGAGTGTGGATCCCAAG | GGTGCTGATGTACCAGTTGG |
| IL-6 | GCTGGAGTCACAGAAGGAGTGG | GGCATAACGCACTAGGTTTGC |
| COX-2 | TAAGTGCGATTGTACCCGGAC | TGTTACGACTGATACCGATGTTT |
| IL-10 | GCTGGACAACATACTGCTAACCG | TCAAATGCTCCTTGATTTCTGGGC |
| Arg-1 | GTGAACACGGCAGTGGCTTTA | AGGCGTTTGCTTAGTTCTGTCTG |
| HIF-1α | AGACAAAGTTCACCTGAGCCTAAT | GGGTTCTTTGCTTCTGTGTCTTC |
| VEGFA | CCTCCGAAACCATGAACTTTCTG | GCGCTGATAGACATCCATGAACT |
| ZO-1 | TCACGCAGTTACGAGCAAGT | TGAAGGTATCAGCGGAGGGA |
| Occludin | TCAGGGAATATCCACCTATCACT TCAG | CATCAGCAGCAGCCATGTACTC TTCAC |
| Claudin-1 | GAAGTGCTTGGAAGACGATG | GAGCCTGACCAAATTCGTAC |
